# The *IDA/IDA-LIKE* and *PIP/PIP-LIKE* gene families in *Arabidopsis*: phylogenetic relationship, expression patterns, and transcriptional effect of the PIPL3 peptide

**DOI:** 10.1093/jxb/erv285

**Published:** 2015-06-10

**Authors:** Ane Kjersti Vie, Javad Najafi, Bin Liu, Per Winge, Melinka A. Butenko, Karina S. Hornslien, Robert Kumpf, Reidunn B. Aalen, Atle M. Bones, Tore Brembu

**Affiliations:** ^1^Department of Biology, Norwegian University of Science and Technology, N-7491 Trondheim, Norway; ^2^Department of Biosciences, University of Oslo, N-0316 Oslo, Norway

**Keywords:** *Arabidopsis*, evolution, gene expression, INFLORESCENCE DEFICIENT IN ABSCISSION, peptide ligand.

## Abstract

Peptide ligands play crucial roles in the life cycle of plants by modulating the innate immunity against pathogens and regulating growth and developmental processes. One well-studied example is INFLORESCENCE DEFICIENT IN ABSCISSION (IDA), which controls floral organ abscission and lateral root emergence in *Arabidopsis thaliana*. IDA belongs to a family of five additional IDA-LIKE (IDL) members that have all been suggested to be involved in regulation of *Arabidopsis* development. Here we present three novel members of the *IDL* subfamily and show that two of them are strongly and rapidly induced by different biotic and abiotic stresses. Furthermore, we provide data that the recently identified PAMP-INDUCED SECRETED PEPTIDE (PIP) and PIP-LIKE (PIPL) peptides, which show similarity to the IDL and C-TERMINALLY ENCODED PEPTIDE (CEP) peptides, are not only involved in innate immune response in *Arabidopsis* but are also induced by abiotic stress. Expression patterns of the *IDA/IDL* and *PIP/PIPL* genes were analysed using *in silico* data, qRT-PCR and GUS promoter lines. Transcriptomic responses to PIPL3 peptide treatment suggested a role in regulation of biotic stress responses and cell wall modification.

## Highlight

This study presents new members of the IDA/IDL and PIP/PIPL families of peptide ligands in *Arabidopsis*, and highlights that family members are linked to stress responses as well as development.

## Introduction

Plants, like all other multicellular organisms, are dependent on cell-to-cell communication for growth and development, as well as for managing and surviving in a challenging and unpredictable environment. Plant cells are linked together by a cellulose wall, and signals between cells passes through plasmodesmata (Gallagher and Benfey, 2005; Kim, 2005) or through ligand-receptor interactions on the cell surface (Shiu and Bleecker, 2001a). Recent evidence indicates that there could be a connection between these two (Stahl and Simon, 2013). For many years, research focused on the classical phytohormones and their abilities to mediate physiological responses, but during the last decade, peptide ligands have emerged as important mediators of cell-to-cell communication in both development and defence (Butenko *et al.*, 2009; Matsubayashi, 2014). Most peptide ligands are translated as prepropeptides and shuttled into the secretory pathway through their N-terminal signal peptide (SP). The SP is removed, followed by further structural modifications to yield the mature peptide ligand (Murphy *et al.*, 2012). Peptide ligands can be divided into two main groups based on these modifications. Cysteine-rich peptides are characterized by an even number of cysteine residues that form intramolecular disulfide bonds upon maturation (Matsubayashi, 2014). Small peptides may on the other hand be generated from the C-terminus of propeptides with a general absence of cysteine residues, and the active peptides may contain post-translational modifications of key amino acids, like tyrosine sulfation, proline hydroxylation and hydroxyproline arabinosylation (Matsubayashi, 2014; Tabata and Sawa, 2014). A large number of genes encoding putative RECEPTOR-LIKE KINASEs (RLKs) and peptide ligands have been identified in the *Arabidopsis* genome (Shiu and Bleecker, 2001b; Lease and Walker, 2006); still, only a few ligands have been characterized and linked to a receptor and a cellular response (Butenko *et al.*, 2009, 2014).

A well-studied peptide of the second category is INFLORESCENCE DEFICIENT IN ABSCISSION (IDA), known to regulate cell separation processes in *A. thaliana* (Aalen *et al.*, 2013). The *ida* mutant fails to undergo floral organ abscission (Butenko *et al.*, 2003), and overexpression of *IDA* leads to premature and ectopic abscission (Stenvik *et al.*, 2006). Twenty amino acids in the C-terminal region, termed EPIP, were shown in genetic experiments to be sufficient to rescue the *ida* phenotype, thus suggesting that EPIP encompasses the active ligand motif of the peptide (Stenvik *et al.*, 2008). IDA mediates its effect through the two LEUCINE-RICH REPEAT RLKs (LRR-RLKs) HAESA (HAE) and HAESA-LIKE 2 (HSL2), as the double knockout *hae hsl2* is phenotypically similar to the *ida* mutant and overexpression of *IDA* is not able to rescue this phenotype (Cho *et al.*, 2008; Stenvik *et al.*, 2008). Moreover, a dodeca hydroxyprolinated peptide within the EPIP domain can activate and bind HSL2, and also activate HAE, although at substantially higher concentration (Butenko *et al.*, 2014). So far, five genes encoding peptides with similarity to IDA, named *IDA-LIKE 1* to *5* (*IDL1* to *5*), have been identified in the *Arabidopsis* genome (Butenko *et al.*, 2003). It has previously been suggested that the *IDL* genes may share a common role in regulating cell separation events, as they are expressed at sites where cell separation occurs, such as during vascular development, stomata formation, root cap sloughing, lateral root emergence and seed shedding (Stenvik *et al.*, 2008; Kumpf *et al.*, 2013).

Bioinformatic tools have been used to identify ~1000 putative peptides in *Arabidopsis*, based on their general features and similarities to known peptides (Lease and Walker, 2010). Currently, only a small fraction of these have been assigned a function. In this paper we present three new members of the *IDA*-*LIKE* family named *IDA-LIKE 6 (IDL6), IDA-LIKE 7 (IDL7*) and *IDA-LIKE 8 (IDL8),* encoding putative proteins with a ligand motif similar to IDA. In addition, we have in parallel with Hou *et al.* (2014) identified a new family of 11 genes termed *PAMP-INDUCED SECRETED PEPTIDES (PIPs*) and *PIP-LIKE (PIPLs*) (Hou *et al.*, 2014) encoding peptides with similarity to IDA/IDLs and the C-TERMINALLY ENCODED PEPTIDEs (CEPs) (Ohyama *et al.*, 2008; Delay *et al.*, 2013; Imin *et al.*, 2013; Roberts *et al.*, 2013). These families can be recognized by the presence of one or both of two C-terminal, conserved core motifs: SGPS, a motif present in the functional peptide of IDA (Butenko *et al.*, 2014), is found in IDA/IDLs and PIP/PIPLs, whereas the GxGH motif located at the extreme C-terminal is common for PIP/PIPLs and CEPs. Interestingly, while the IDA/IDL and CEP members characterized so far are involved in developmental processes, we show that the PIP/PIPL peptides are involved in stress responses.

## Materials and methods

### Identification of IDA/IDL and PIP/PIPL family genes and phylogenetic analyses

Full-length protein sequences and the conserved C-terminal domain of IDA and IDL1-5 were used in TBLASTN searches against expressed sequence tag (EST), genomic and non-redundant nucleotide databases at NCBI (Altschul *et al.*, 1997). In order to further investigate the presence of *IDA/IDL* family members in other plant species, similar BLAST searches were performed on the Phytozome v9.1 genome (Goodstein *et al.*, 2012) and OneKP EST (https://sites.google.com/a/ualberta.ca/onekp/ accessed 28 May 2015) databases. Protein alignments were made using the ClustalX programme (Larkin *et al.*, 2007) and later manually refined with GeneDoc (Nicholas *et al.*, 1997). Neighbour-joining (N-J) trees were produced from the protein alignments using the N-J method (Saitou and Nei, 1987) and Kimura’s correction for multiple substitutions as implemented in the ClustalX programme. In total 1000 bootstrap trials were run on the N-J tree. Maximum-likelihood (ML) analysis of the full-length IDA/IDL and PIP/PIPL protein alignments were performed using the RAxML programme (Stamatakis, 2006) with the PROTGAMMABLOSUM62 substitution model and running 1000 bootstrap replicas. BLOSUM substitution matrices were used in both ML and N-J analyses. Trees were visualized using TreeView 1.6.6 (Page, 2002) and refined in Adobe Illustrator CS6. SP sequences were identified through SignalP 4.0 (Petersen *et al.*, 2011) (http://www.cbs.dtu.dk/services/SignalP/ accessed 28 May 2015). Protein sequence motif visualization was done using WebLogo (Crooks *et al.*, 2004; Schneider and Stephens, 1990). Analysis of gene duplication events and identification of syntenic regions was done by screening the 40 nearest protein coding genes flanking each of the *IDA/IDL-PIP/PIPL* gene loci for other closely related genes located next to the *IDA/IDL* and *PIP/PIPL* genes. Each region was analysed by BLASTP searches, and a custom-made Perl script was used to parse BLAST tables and identify high scoring proteins (included in the top 5 score list) that had corresponding genes mapping to *IDA/IDL-PIP/PIPL* genomic regions.

### Plant material

Seeds of the *Arabidopsis thaliana* ecotype Col-0 (N1092) were obtained from the European Arabidopsis Stock Centre (NASC, Nottingham, UK).

The five *pIDL:GUS* constructs were made using Gateway technology. The promoters included 1555, 1864, 1908, 1980 and 2020bp upstream of the ATG start codon of *IDL1* to *IDL5*, respectively (Stenvik *et al.* 2008).

### Plant growth conditions and plant tissue collection for expression analysis during development

Seeds of Col-0 ecotype were surface sterilized and sown on half-strength MS plates supplemented by 2% (w/v) sucrose at a density of 44 seeds per Petri dish (14cm diameter) and stratified for 3 d at 4°C before being transferred to a controlled *in vitro* growth room under a 16h light (70 µmol m^-2^ sec^-1^): 8h dark photoperiod at 22°C. At stage 1.10 (Boyes *et al.*, 2001), plants were transferred to soil and grown further in a controlled growth chamber (VB1514, Vötsch Industrietechnik, Balingen, Germany) under the same light conditions at 22°C until the end of the experiment.

Tissue was harvested at different growth stages as defined by Boyes *et al.* (2001). For stages 1.0, 1.06 and 1.10, whole plantlets were harvested from *in vitro* cultivation medium. At the later stages roots, rosette leaves, cauline leaves, stem, inflorescences and siliques were harvested separately. All material was immediately flash frozen in liquid nitrogen upon harvesting and stored at −80°C until further processing. Three biological replicates were harvested, where each replicate consisted of plant material pooled from eight Petri dishes (stage 1.0), four Petri dishes (stages 1.06 and 1.10) and five plants (stages 6.00 and 8.00), respectively.

### Stress treatments

All treatments were conducted on 2-week-old wild-type seedlings corresponding to growth stage 1.06 (Boyes *et al.*, 2001) unless otherwise stated. Seeds of Col-0 ecotype were surface sterilized and sown out on half-strength MS plates supplemented by 2% (w/v) sucrose at a density of 20 seeds per Petri dish (14cm diameter). For chitin, cycloheximide (CHX) and anisomycin treatments, seedlings were sprayed with 10 µg/ml chitin, 10 µg/ml CHX or 15 µg/ml anisomycin in MilliQ (MQ) water added 0.02% Silwet L-77 (Lehle Seeds) and vacuum infiltrated at 20 inches Hg for 1min. As control, plants were treated with MQ water added 0.02% Silwet L-77 and vacuum infiltrated at 20 inches Hg for 1min. Seedlings were incubated 1h (chitin) and 6h (CHX and anisomycin) after treatment under normal growth conditions before harvesting. Salt treatment was conducted in 24 well plates (1 seed per well) containing 1ml liquid half-strength MS and 2% (w/v) sucrose. At stage 1.06 (Boyes *et al.*, 2001), the medium was replaced with liquid half-strength MS supplemented with 2% sucrose (w/v) and NaCl (150mM). Control plants were placed in fresh half-strength MS medium [2% sucrose (w/v)] without NaCl. The seedlings were treated for 6h before harvesting. For all stress experiments, three biological replicates were harvested, each replicate consisting of plant material pooled from three Petri dishes. *Brevicoryne brassicae* treatments were conducted as described in Kuśnierczyk *et al.* (2011).

### Peptide treatments for microarray analyses

Peptides of the putative ligand motif of PIPL3 [LSSAGERMHTMASG(HYP)SRRGAGH, where HYP is hydroxyproline] and a mock peptide (LSPGKNLSAPGRVGSNPFTKLRGS) were synthesized with a purity of >95% by Biomatik (Cambridge, Canada). Seeds of Col-0 ecotype were surface-sterilized and sown out on half-strength MS plates at a density of 20 seeds per Petri dish (14cm diameter), and stratified for 3 d at 4°C. Plates were grown under a 16h photoperiod (70 µmol m^-2^ s^-1^) at 22°C for 2 weeks. Seedlings were sprayed with an aqueous peptide solution (100nM) supplemented with 0.02% silwet L-77 (Lehle Seeds, UK). Whole rosettes were collected 3h after treatment, snap-frozen in liquid nitrogen, and stored at −80°C.

### RNA extraction and cDNA synthesis

100mg frozen plant tissue each from four biological replicas were homogenized using TissueLyser II (Qiagen, Hilden, Germany) for 2×2min at 25 Hz. Total RNA was extracted with the Spectrum Plant Total RNA kit (Sigma-Aldrich, Saint Louis, USA) as described by the supplier, but with lysis solution being added to the plant tissue between the two disruption cycles. An on-column DNase digestion was performed using the RNase-Free DNase Set (Qiagen, Hilden, Germany). Total RNA was quantified using NanoDrop ND-1000 (Nanodrop, Delaware, USA) and RNA quality was verified by formaldehyde gel electrophoresis. RNA was stored at −80°C until used.

cDNA synthesis was performed on 1 µg total RNA using the QuantiTect Reverse Transcription Kit (Qiagen, Hilden, Germany), following the supplier’s instructions. cDNA samples were diluted 10-fold before use in qRT-PCR reactions.

### Quantitative real-time PCR

Quantitative real-time PCR (qRT-PCR) was performed on a LightCycler 480 using the LightCycler 480 SYBR Green I Master kit (Roche Applied Science, Mannheim, Germany), with PCR parameters as recommended by the supplier: pre-incubation was performed at 95°C for 5min, followed by 50 amplification cycles, each consisting of 10 s denaturation at 95°C, 10 s annealing at 55°C and 10 s elongation at 72°C. *TIP41-LIKE* (At4g34270) was used as reference gene (Czechowski *et al.*, 2005) for the stress and developmental analyses, and *CYP71A13* (At2g30770) was used as negative RT control. PCR efficiencies and C_t_ values were calculated by linear regression using the LinRegPCR software (Ramakers *et al.*, 2003; Ruijter *et al.*, 2009), and mean PCR efficiency was calculated for each pair of primers. C_t_-values and PCR efficiencies were then imported into the REST 2008 software (Pfaffl *et al.*, 2002) to calculate the statistical significance of differences in expression levels upon various treatments. Primers used are listed in Supplementary Table S1.

### Microarray and statistical analysis

Genome-wide expression analysis was performed using the *Arabidopsis* (V4) Gene Expression Microarray 4×44K (Agilent Technology, USA) as described by the supplier’s manual: total RNA (~0.2 µg) was reverse transcribed, amplified and labelled using the Low Input Quick Amp Labeling Kit, two-colour protocol, (Agilent p/n 5190-2306) (Agilent Technologies, USA). Hybridization was performed with the Gene Expression Hybridization Kit (Agilent p/n 5188–5242). 825ng cRNA from both mock-treated plants and PIPL3 peptide-treated plants were used. The cRNA mixture was fragmented and hybridized on *Arabidopsis* (V4) Gene Expression Microarray 4×44K arrays in a rotary oven at 65°C for ~15h. cRNA from the mock- and PIPL3-treated plants were alternately labelled with Cy3 or Cy5, which makes it possible to assess dye bias effects during the statistical analysis. The slides were washed with Gene Expression Wash Buffer 1 (Agilent p/n 5188–5325), Gene Expression Wash Buffer 2 (Agilent p/n 5188–5326), acetonitrile (VWR International) and Stabilization and Drying Solution (Agilent Technologies) according to the manufacturer’s instructions). The slides were scanned at 5 µm resolution on an Agilent DNA microarray scanner (Agilent Technologies). The image files were analysed with the Agilent Feature Extraction Software.

Prior to the statistical analysis spots from control spikes, landmarks and genes with low expression (absent) were filtered out. The data were analysed using the limma package (Smyth, 2005) and the R statistical data analysis programme package (R 2.10.1). No background subtraction was performed, and data were normalized using the Global Loess Normalization method. Benjamini and Hochberg’s method to control the false discovery rate (FDR) was used to identify differentially regulated genes (Benjamini and Hochberg, 1995). Genes with dye bias effects were removed and genes with an adjusted *P*-value of less than 0.05 were regarded as significantly differentially expressed. The study is MIAME compliant. Raw data has been deposited in GEO (accession GSE66201).

### GO analysis

Gene ontology (GO) annotation analysis was performed using the Cytoscape 3.1.0 (Smoot *et al.*, 2011) plug-in Bingo 3.0.2 (Maere *et al.*, 2005). Over-represented categories were identified using a hypergeometric test with a significance threshold of 0.05 after Benjamini and Hochberg’s FDR correction (Benjamini and Yekutieli, 2001) using the whole annotated genome as the reference set.

### Histochemical GUS assays

Histochemical GUS assays were performed as described by Butenko *et al.* (2003).

Sequence data from this article can be found in the GenBank/EMBL data libraries under accession numbers NM_105550.1 (IDA), NM_113464.2 (IDL1), NM_001085327.2 (IDL2), NM_001085091.1 (IDL3), NM_001084711.1 (IDL4), AY642386.1 (IDL5), NM_120612.1 (IDL6), AK118348.1 (IDL7), AK221754.1 (IDL8), NM_118988.2 (PIP1), NM_119892.1 (PIP2), NM_127891.1 (PIP3), NM_103867.2 (PIPL1), NM_111484.1 (PIPL2), NM_119893.1 (PIPL3), NM_001125892.1 (PIPL4), CB254609.1 (PIPL5), EF183199.1 (PIPL6), NC_003075.7 (PIPL7), NC_003076.8 (PIPL8), NM_103641 (CEP1), NM_148611.1 (CEP2), NM_127908.1 (CEP3), NM_201876 (CEP4), NM_126080.1 (CEP5), NM_114921.1 (CEP6), NC_003076.8 (CEP7 and CEP8), NM_114921.1 (CEP9), NC_003070.9 (CEP11), NC_003071.7 (CEP12), NM_101556.3 (CEP13), NM_102669.3 (CEP14) and NM_129615.3 (CEP15).

## Results

### Identification of *IDL* and *PIP/PIPL* genes in *Arabidopsis*

**Fig. 1.**
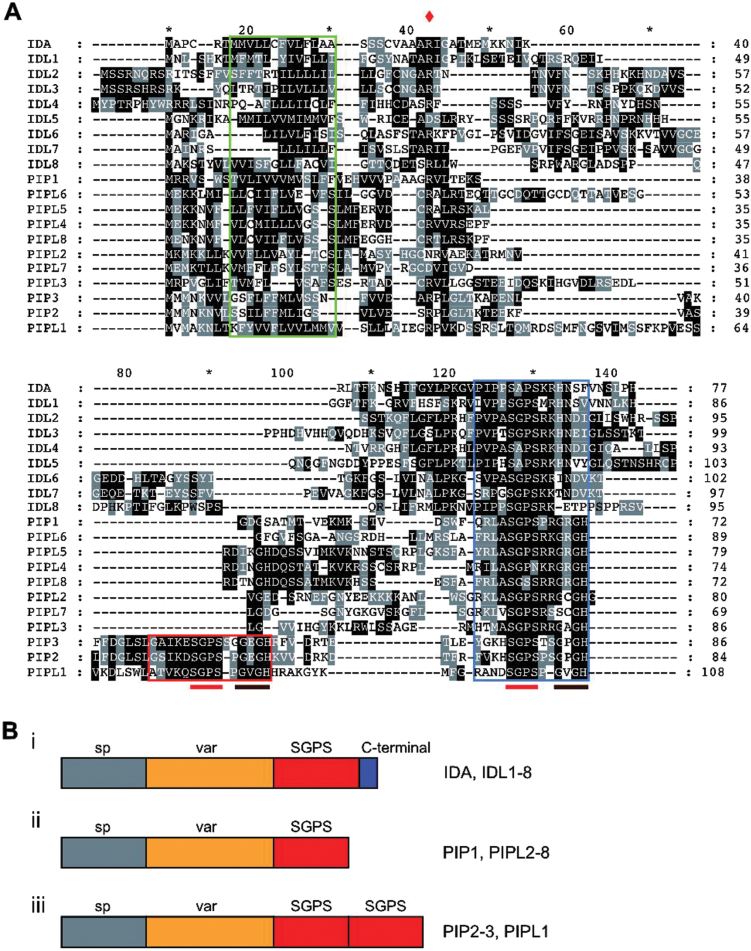
The IDA/IDL and PIP/PIPL peptide families. (A) Protein alignment based on full-length sequences of the IDA/IDL/PIP/PIPL proteins. The green box indicates the SP, the blue box indicates the C-terminal putative peptide ligand motif and the red box indicates the second ligand motif identified in PIP2, PIP3 and PIPL1. The conserved arginine following the predicted SP is marked by a red diamond. The SGPS and GxGH motifs are indicated by red and brown bars below the alignment, respectively. (B) Schematic representation of the proteins in the IDA/IDL and PIP/PIPL peptide families. Grey boxes (marked with ‘sp’) represent the SP sequences identified by SignalP 4.1, orange boxes (marked with ‘var’) indicates a variable region with little homology, and red boxes represents the conserved, C-terminal EPIP domain known to be the active part of the IDA peptide indicated by the conserved core motif SGPS. The IDA and IDL proteins possess a C-terminal, variable sequence (i). This non-conserved sequence is not found among the PIP/PIPLs (ii). PIP2, PIP3 and PIPL1 contain two tandem SPGS motifs (iii).

Six members of the *IDA* gene family have been identified in *Arabidopsis* to date: *IDA* and *IDL1* to *IDL5*. All members of the *IDA* gene family are intronless and encode small proteins (<110 amino acids) characterized by an N-terminal secretory SP, a variable region and a C-terminal, conserved region (Butenko *et al.*, 2003). Through database searches, three new *IDL* genes were found (Table 1; Fig. 1A; Supplementary Dataset S1). In addition, we identified 11 genes with similarity to the *IDLs*. During preparation of this paper an article was published presenting these genes as a family encoding secreted PAMP-INDUCED PEPTIDES (PIPs) and PIP-LIKE (PIPL) peptides (Hou *et al.*, 2014) (Table 1; Fig. 1A; Supplementary Dataset S1). For all members the N-terminal SP contains a stretch of aliphatic residues typical of secreted proteins (Fig. 1A, green box). This motif is followed by a conspicuously conserved arginine residue (Fig. 1A, red diamond). The C-terminal is characterized by the conserved core motif S(G,A,V)PS (hereafter called the SGPS motif) conserved in both IDLs and PIP/PIPLs (Fig. 1A, blue box). The SGPS motif in IDL proteins is followed by four highly conserved residues [(R/K)(R/K)HN] followed by up to 13 additional less conserved residues (Fig. 1A, Bi). The PIP/PIPL proteins lack the variable region C-terminal to the SGPS motif that is found for the IDL proteins (Fig. 1A, Bii, iii). Three of the PIP/PIPLs (PIP2, PIP3 and PIPL1) contain two SPGS motifs in a tandem orientation at the C-terminal (Fig. 1A, Biii), as identified by Hou *et al.* (2014).

**Table 1.** The *IDA/IDL* and *PIP/PIPL* gene families in Arabidopsis

**Gene name**	**Locus**	**Accession number**	**Signal peptide aa**	**Protein aa**
*IDA*	At1g68765	NM_105550.1	26	77
*IDL1*	At3g25655	NM_113464.2	27	86
*IDL2*	At5g64667	NM_001085327.2	36	95
*IDL3*	At5g09805	NM_001085091.1	32	99
*IDL4*	At3g18715	NM_001084711.1	36	93
*IDL5*	At1g76952	AY642386.1	27	103
*IDL6*	At5g05300	NM_120612.1	24	102
*IDL7*	At3g10930	AK118348.1	21	97
*IDL8*	At5g02591^a^	AK221754.1	22	95
*PIP1*	At4g28460	NM_118988.2	30	72
*PIP2*	At4g37290	NM_119892.1	24	84
*PIP3*	At2g23270	NM_127891.1	19	86
*PIPL1*	At1g49800	NM_103867.2	27	108
*PIPL2*	At3g06090	NM_111484.1	22	79
*PIPL3*	At4g37295	NM_119893.1	22	86
*PIPL4*	At5g43066	NM_001125892.1	21	74
*PIPL5*	At5g43068^*a*^	CB254609.1	21	79
*PIPL6*	At1g47178^*a*^	EF183199.1	22	88
*PIPL7*	At4g11402^*a*^	NC_003075.7 (w/6941763-6941972)^*b*^	23	69
*PIPL8*	At5g43064^*a*^	NC_003076.8 (w/17282272-17282490)^*b*^	21	72

^*a*^ Preliminary AtID from TAIR

^*b*^ Chromosomal coordinates of cds

**Fig. 2.**
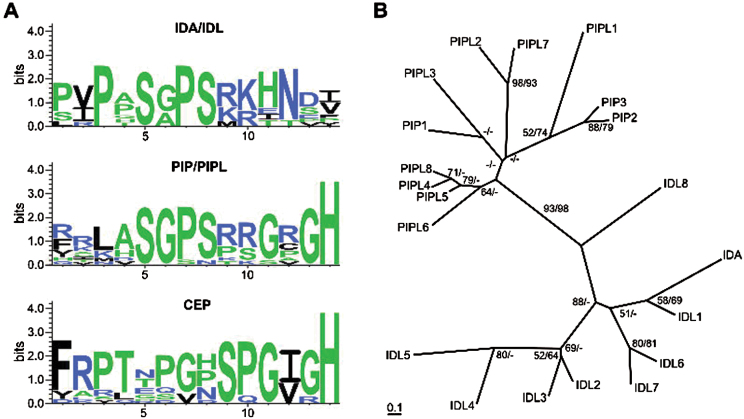
Phylogenetic relationship between the IDA/IDL and PIP/PIPL peptides. (A) Sequence logo representation of the conserved C-terminal of IDA/IDL, PIP/PIPL and CEP peptides. (B) N-J and ML trees were constructed based on the protein alignment of the IDA/IDL and PIP/PIPL families shown in Fig. 1A. The N-J trees are shown. The overall topologies for the N-J and ML trees are the same. Bootstrap confidence values above 50% for N-J (first value) and ML (second value) are shown in the tree.

Two studies of the CEP family of small peptides, consisting of 15 members, have recently been published (Delay *et al.*, 2013; Roberts *et al.*, 2013). Two of the CEP family members, CEP13 and CEP14, show similarity to the PIP/PIPLs. A sequence consensus logo of the SGPS motif and surrounding residues was made for the IDL and PIP/PIPL families as well as for the CEP family (Fig. 2A). The SGPS motif (position 4–7) is conserved in IDLs and PIP/PIPLs. A second conserved motif (GxGH, where x is any amino acid) was seen at the C-terminal end of all PIP/PIPLs. The SGPS motif was not found in the CEPs; however, the family was characterized by the C-terminal SPG(I/V)GH sequence, which resembles the C-terminal end of the PIP/PIPLs. Thus, the putative ligand domain of the PIP/PIPLs shares features with both IDLs and CEPs.

### Phylogenetic analysis of the IDA/IDL and PIP/PIPL families

In order to examine the relationship of the *IDA/IDL* and *PIP/PIPL* gene families in *Arabidopsis*, a phylogenetic analysis on the full-length protein sequences of all IDL and PIP/PIPL members was performed. Two methods were used: a distance-matrix method combined with the N-J algorithm as implemented in the ClustalX programme, and a ML method using a gamma model and the RAxML programme (Fig. 2B). The resulting tree topologies from the two analyses were highly similar (data not shown), although bootstrap confidence values were a bit lower for the ML analysis. The analysis shows that the IDA/IDL and PIP/PIPL families split into separate branches with a high level of bootstrap confidence, even though the highly variable central part of the proteins is included in the analysis. Due to the high sequence divergence of these genes, not all branches in the tree are supported with high confidence levels. However, the IDA/IDL cluster can be divided into two subgroups: one containing IDL2, IDL3, IDL4 and IDL5 and the other containing IDA, IDL1, IDL6 and IDL7. Identical ML and N-J tree topologies support this division. Of the IDL proteins, IDL8 can be regarded as an outlier, and does not cluster well with any of the other IDLs. The PIP/PIPL proteins can be broadly divided into two groups: those with a single SGPS motif (PIP1 and PIPL2–PIPL8) and those with two SGPS motifs (PIP2, PIP3 and PIPL1).

A phylogenetic analysis (using the ML method) was also performed on a full-length protein alignment of the IDA/IDL and PIP/PIPL families as well as the CEP family (Supplementary Fig. S1). CEP9 was not included in the analysis due to its aberrant length and number of peptide motifs (five). The IDA/IDL family was clearly separated from the PIP/PIPL and CEP families with a high bootstrap confidence value (100%). Furthermore, CEP13, CEP14 and CEP15 [defined as group II CEPs by Delay *et al.* (2013) and Roberts *et al.* (2013) formed a clade with significant bootstrap values.

To further study the evolutionary relationship between the *IDA/IDL* and *PIP/PIPL* genes, the region surrounding the *IDA/IDL-PIP/PIPL* gene loci were analysed for ancient chromosomal or gene duplications. The results are summarized in Supplementary Table S2. The chromosomal localization of the genes are shown in Supplementary Fig. S2. *PIPL5*, *PIPL4* and *PIPL8* are organized in tandem repeats, as are *PIP2* and *PIPL3*. *IDA* and *IDL1* are likely the result of a recent duplication event, as seven of the 40 genes flanking *IDA* and *IDL1* are closely related to each other. This is the case for several other genes in the *IDA/IDL* family as well; *IDL2* and *IDL3* share 13–15 common neighbouring genes, as previously noted by Stenvik *et al.* (2008), while *IDL6* and *IDL7* shares six. *PIPL6* have five neighbouring genes with corresponding homologues surrounding the *PIPL5-PIPL4-PIPL8* loci and *PIP3* shares four to eight genes with *PIPL3-PIP2*, depending on the region used for BLAST search.

The regions flanking *IDL5* do not share any closely related genes located within the other *IDA-PIPL* regions, while *IDL8* and *PIPL6* only share a few. This lack of synteny suggests that these genes have evolved through mechanisms other than tandem gene duplication, such as movement via RNA intermediates like retrotransposable elements.

**Fig. 3.**
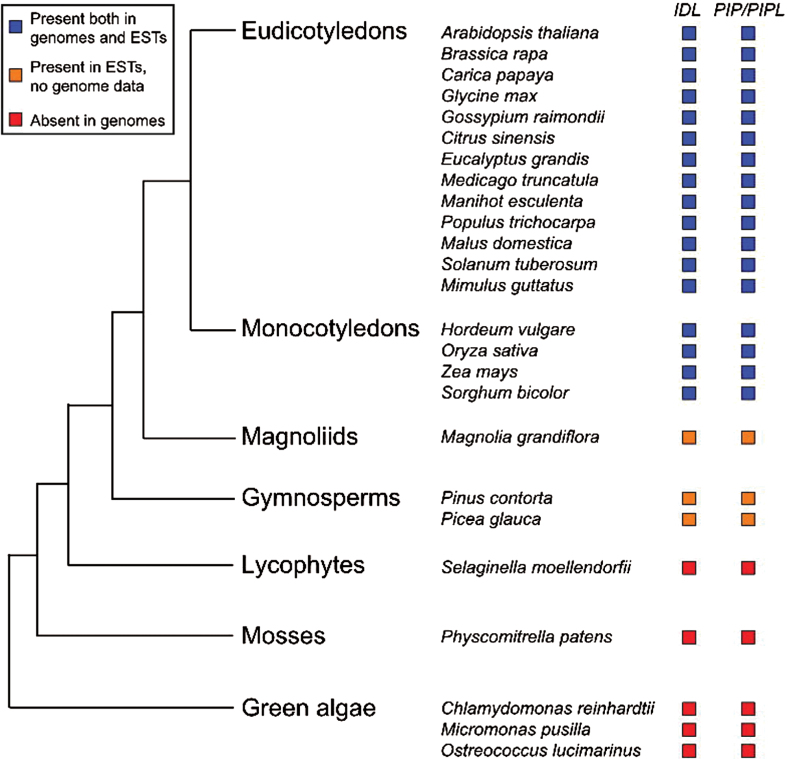
Distribution of *IDA/IDL* and *PIP/PIPL* genes within Viridiplantae. The conserved C-terminal domain of IDA/IDL and PIP/PIPL proteins were used in TBLASTN searches against the NCBI (genomes and ESTs), Phytozome v9.1 (genomes) and OneKP (ESTs) databases. The tree was adapted from Phytozome (Goodstein *et al.*, 2012) and Delay *et al.* (2013).

BLAST searches for *IDA/IDL* and *PIP/PIPL* family genes within the Viridiplantae were performed using full-length protein sequences as well as mature peptide sequences of *Arabidopsis* proteins. The searches showed that genes encoding both peptide families are present in seed plants, but absent in lycophytes (*Selaginella*), mosses (*Physcomitrella*) and green algae (Fig. 3). This distribution is similar to the one previously reported for CEPs (Delay *et al.*, 2013; Roberts *et al.*, 2013).

### Expression patterns of *IDA/IDL* and *PIP/PIPL* genes during development

**Fig. 4.**
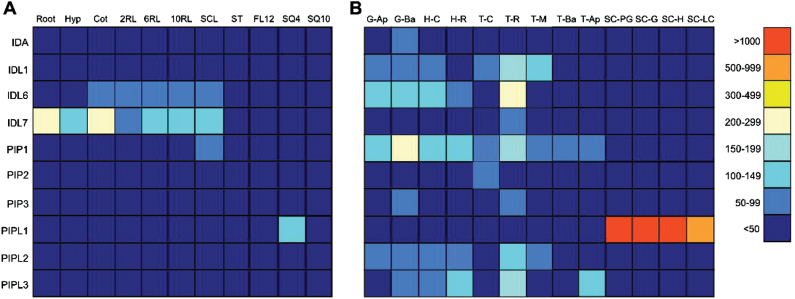
Developmental expression patterns of *IDA/IDL* and PIP/*PIPL* genes based on *in silico* data. (A) Expression in different vegetative tissues during development. (B) Expression during embryo and seed development (Casson *et al.*, 2005). All data was obtained from the *Arabidopsis* eFP browser at the Bio-Array Resource database (Winter *et al.*, 2007). The arithmetic expression values are given next to the colour scale. Hyp, hypocotyl; Cot, cotyledon; 2RL, second rosette leaf; 6RL, sixth rosette leaf; 10RL, tenth rosette leaf; SCL, senescing leaves; ST, stem; FL12, flower stage 12; SQ4, silique position 4; SQ10, silique position 10; G-Ap, globular stage apical; G-Ba, globular stage basal; H-C, heart stage cotyledon; H-R, heart stage root; T-C, torpedo stage cotyledon; T-R, torpedo stage root; T-M, torpedo stage meristem; T-Ap, torpedo stage apical; T-Ba, torpedo stage basal; SC-PG, seed coat preglobular stage; SC-G, seed coat globular stage; SC-H, seed coat heart stage; SC-LC, seed coat linear cotyledon.

Since the expression pattern of a gene may provide an indication of its function, we conducted an *in silico* analysis of the transcription levels of the genes included on the Affymetrix ATH1 microarrays (*IDA, IDL1, IDL6, IDL7, PIP1*-*PIP3* and *PIPL1-PIPL3*) during plant development (Schmid *et al.*, 2005), obtaining data from the eFP Browser (Winter *et al.*, 2007). These genes are expressed at low levels both at post-germination stages and during embryogenesis (Fig. 4). However, *IDL1*, *IDL6*, *PIP1* and *PIPL3* are expressed in several embryonic tissues [data obtained from Casson *et al.* (2005)], with the highest expression found in root primordia during the torpedo stage. *PIP1* is also expressed in basal tissue during the globular stage. *PIPL1* is the most strongly expressed gene during seed development. In contrast to the rest of the genes, *PIPL1* is not expressed in the embryo, but shows high expression levels in the seed coat during early stages of seed development.

Although the publicly available gene expression databases provide valuable information about the *IDA/IDL* and *PIP*/*PIPL* genes, it is very incomplete. Ten of the genes (*IDL2–5*, *IDL8, PIPL4–8*) are not included on the Affymetrix ATH1 microarrays used to generate these data. To fully assess the expression pattern of all the genes, qRT-PCR was performed on RNA isolated from Col-0 ecotype tissue harvested at various growth stages during the plant life cycle, as described by Boyes *et al.* (2001). Since none of the genes in the family contain introns, a control for genomic contamination was included in the analysis, using primers for *CYP71A13* spanning the third intron in this gene.

**Fig. 5.**
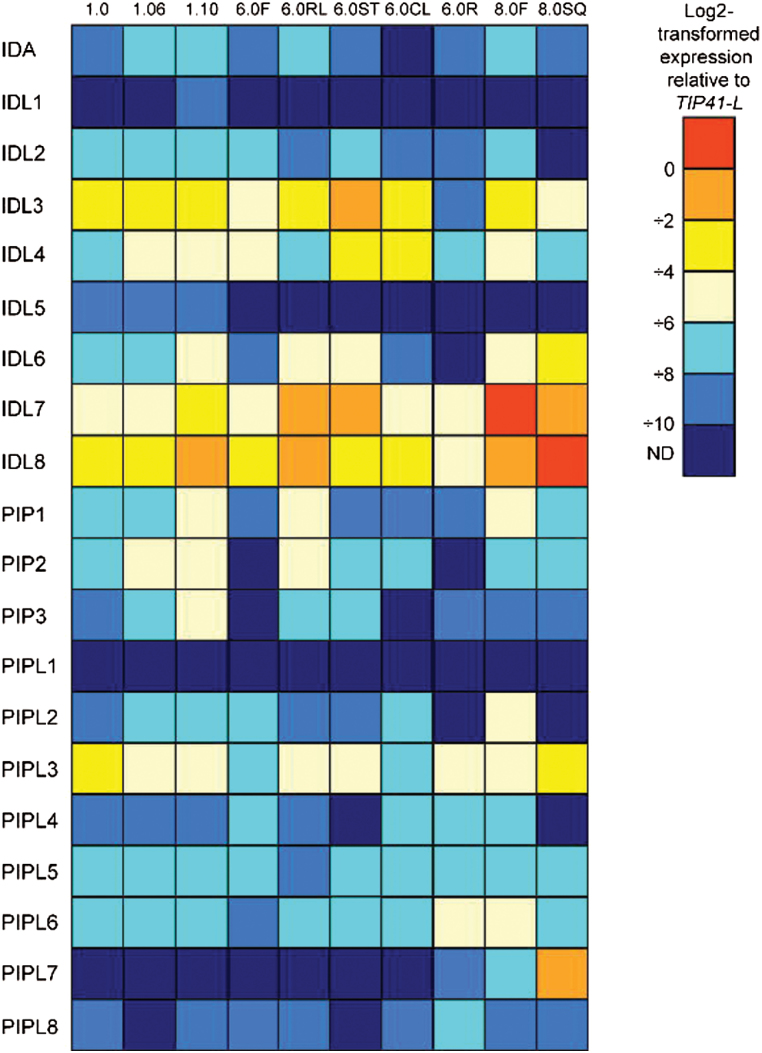
Developmental expression patterns of *IDA/IDL* and PIP/*PIPL* genes based on quantitative real-time PCR. The different developmental stages are annotated according to Boyes *et al.* (2001). F, flower; RL, rosette leaf; ST, stem; CL, cauline leaf; R, root; SQ, silique; ND, not detected. The expression levels (log_2_-transformed) relative to *TIP41-LIKE* are given next to the colour scale. *n*=3.

The expression levels of the *IDA/IDL* and *PIP/PIPL* genes under normal growth conditions were in general very low (Fig. 5); for some of the genes (*IDL1*, *IDL5* and *PIPL1*) hardly any transcripts were detected. The highest transcript levels were found at the later stages of *Arabidopsis* development (i.e. *IDL6*, *IDL7* and *IDL8*), indicating a possible role for these genes during seed development or senescence. Other genes, like *IDL3* and *PIPL3*, were weakly expressed during all stages.

### GUS expression analyses of *IDL* genes

**Fig. 6.**
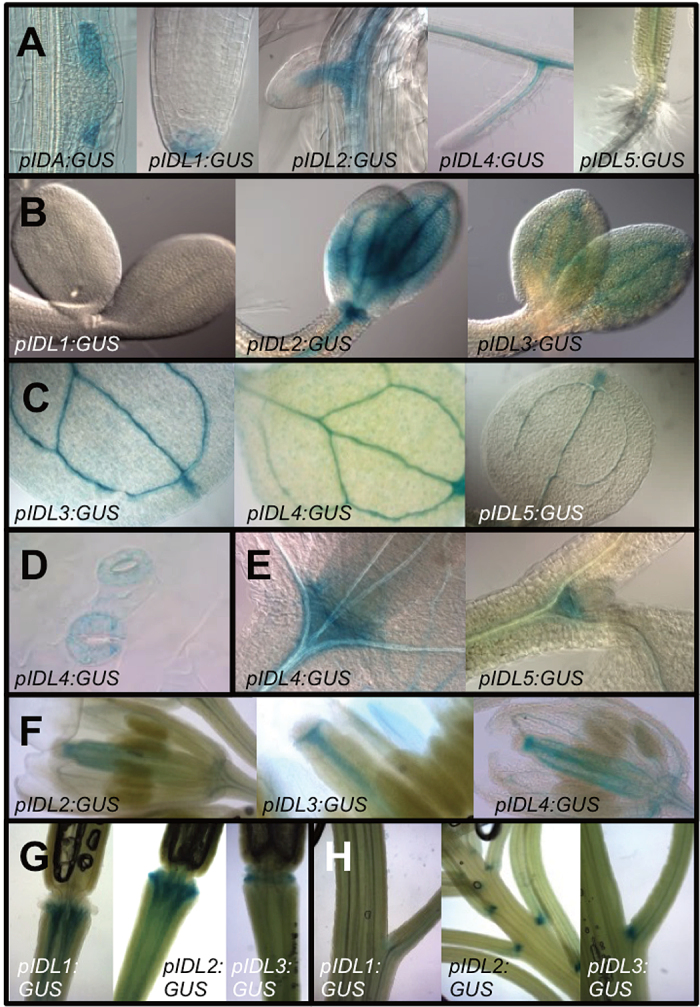
Histochemical analysis of promoter:GUS expression using the promoters of *IDA* and *IDL1 to IDL5* genes. (A) *pIDA:GUS* is expressed during lateral root emergence in endodermis (63× magnification), cortex and epidermis cells; *pIDL1:GUS* in columella root cap cells (40×); *pIDL3:GUS* and *pIDL4:GUS* in the vasculature and *pIDL5:GUS* next to the hypocotyl (20×). (B) *pIDL2:GUS* and *pIDL3:GUS* are, in contrast to *pIDL1:GUS,* expressed in the cotyledons 3 d after germination (40×). (C) *pIDL3:GUS, pIDL4:GUS* and *pIDL5:GUS* are expressed in the vasculature of the first true leaves, including the hydathodes (20×). (D) *pIDL4:GUS* is expressed in the guard cells (40×); (E) *pIDL4:GUS* and *IDL5:GUS* are expressed in the shoot apical meristem (40×), (F) *pIDL2:GUS*, *pIDL3:GUS* and *pIDL4:GUS* are expressed in the vasculature of flowers at developmental stage 6.0F (20×). (G) *pIDL1:GUS, pIDL2:GUS* and *pIDL3:GUS* are expressed in the vasculature in the abscission zone region after the floral organs have been shed (20×). (H) *pIDL2:GUS* and *pIDL3:GUS* are in contrast to *pIDL1:GUS* expressed in vestigial abscission zones at the base of pedicels and branches (20×).

To further investigate the expression pattern of the different *IDL* genes during early *Arabidopsis* development, plants expressing *promoter:GUS* reporter constructs for *IDA* and *IDL* genes (*pIDA*/*pIDL:GUS*) were investigated from germination up until 14 d after germination (Supplementary Table S3). *pIDA*:*GUS* expression was observed in the cortex and epidermal cells overlaying the lateral root primordia in accordance with the recently reported function in cell separation allowing lateral root emergence (Fig. 6A; Kumpf *et al.*, 2013). *pIDL1*:*GUS* had a specific pattern of expression in the columella root cap cells of the primary root (Fig. 6A), where cells undergo cell separation during root cap sloughing, which allows the primary root to penetrate the soil (del Campillo *et al.*, 2004). After germination, *pIDL2:GUS, pIDL4:GUS* and *pIDL5:GUS* were expressed in the vascular tissue of the primary and lateral roots (Fig. 6A). Consistent with the expression in leaves identified by qRT-PCR (Fig. 5), the *pIDL:GUS* constructs were all expressed in the vascular tissue of the expanding cotyledons and/or primary leaves (Fig. 6B, C), and *IDL4* expression was also observed in the guard cells (Fig. 6D) (Stenvik *et al.*, 2008). The *IDL promoter:GUS* constructs were all expressed in the shoot apical meristem region, here represented by *IDL4* and *IDL5* in Fig. 6E. Expression was then investigated in various tissues at later developmental stages, as described by Boyes *et al.* (2001). Previous studies have shown that *IDA*, *IDL2*, *IDL3* and *IDL4* are expressed in the floral abscission zone (Stenvik *et al.*, 2008). None of the genes were expressed in the abscission zone region at the time points investigated by qRT-PCR, although expression was detected in the vasculature of the developing floral organs (Fig. 6F). However, *IDL1*, *IDL2* and *IDL3* showed expression in the abscission zone at later stages, when the floral organs are already abscised (Fig. 6G). In addition, the same genes showed expression in the vestigial abscission zones of the pedicel region (Fig. 6H).

### A subset of *IDA/IDL* and *PIP/PIPL* genes are induced by biotic and abiotic stress

**Fig. 7.**
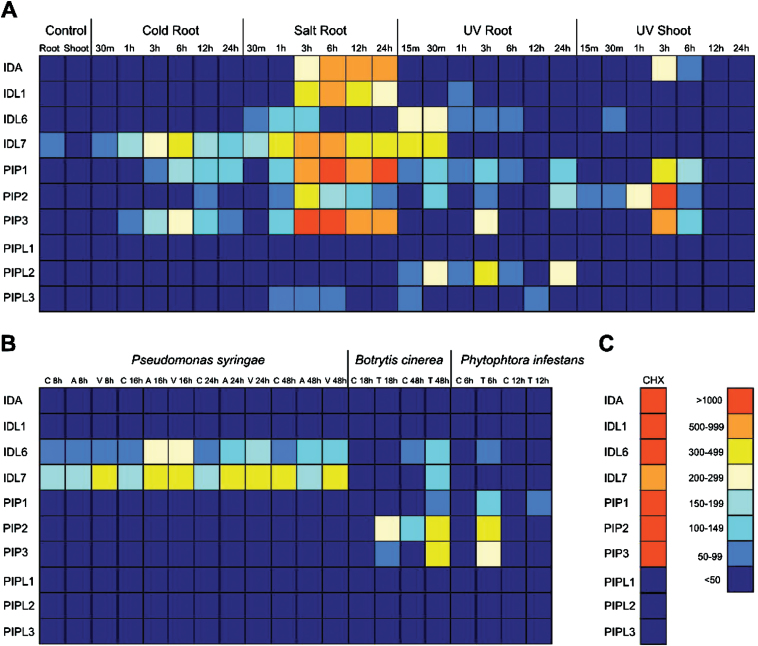
Stress-induced expression of *IDA/IDL* and *PIP/PIPL* genes based on *in silico* data. (A) Abiotic stress. Cold root, root tissue collected from cold-treated seedlings (continuous 4°C); salt root, root tissue collected from salt-treated seedlings (150mM NaCl); UV root/shoot, root and shoot tissue collected from UV-treated seedlings (15min treatment in UV-B field). (B) Biotic stress. *Pseudomonas syringae* half-leaf infiltration: C, control (10mM MgCl_2_); A, avirulent *P. syringae* ES4326 avrRPt2; V, virulent *P. syringae* ES4326. *Botrytis cinerea* treatments: C, control (potato dextrose broth); T, treated (*B. cinerea* 5×10^5^ spores/ml). *Phytophthora infestans* treatments: C, control (water); T, treated (*Phytophthora infestans* 10^6^ spores/ml). (C) Cycloheximide (CHX) treatment (10 μM CHX, 3h). All data was obtained from the *Arabidopsis* eFP browser at the Bio-Array Resource database (Winter *et al.*, 2007). The arithmetic expression values are given next to the colour scale.

In general, the members of the *IDA/IDL* and *PIP/PIPL* families were found to be only weakly expressed under normal growth and development, so the response of *IDA/IDL* and *PIP/PIPL* genes to different abiotic and biotic stresses were therefore investigated (Fig. 7). Figure 7A summarizes the *in silico* analysis of abiotic stresses (Kilian *et al.*, 2007). Cold stress induces expression of *IDL7*, *PIP1* and *PIP3* in roots, whereas UV induces eight out of 11 of the *IDA/IDL/PIP/PIPL* genes present on the Affymetrix ATH1 microarrays. The highest expression is observed in roots during salt stress. *IDA*, *IDL1*, *IDL7*, *PIP1* and *PIP3* are especially highly induced upon such stress, with expression levels up-regulated 500–1000 times compared to the control. *PIPL1* is not expressed during any of the specified treatments. Biotic stress and treatments with elicitors (Fig. 7B; Supplementary Fig. S3) induces fewer genes than abiotic stress. Treatments with both virulent and avirulent strains of the biotrophic pathogen *Pseudomonas syringae* induce *IDL6* and *IDL7* expression. It should be noticed that expression of *IDL6* is induced 1h after treatments with the pathogen-derived elicitors flg22, HrpZ and NPP1. *PIP1* is up-regulated upon treatment with NPP1 and HrpZ (Supplementary Fig. S3). The necrotrophic pathogens *Botrytis cinerea* and *Phytophtora infestans* both induce expression of *PIP2* and *PIP3*. This is in accordance with data recently published by Hou *et al.* (2014), showing that PIP1, PIP2 and PIP3 are involved in amplification of the immune response. *IDL6* and *IDL7* are in general induced at earlier time points than the rest of the genes (Fig. 7; Supplementary Fig. S3). Transcripts of *IDL6* and *IDL7* are detected as early as 15min post UV exposure, while *PIPL2* and *PIP3* are detected 30min (*PIPL2*) and 3h (*PIP3*) after exposure (Fig. 7A). Data obtained from Genevestigator (Hruz *et al.*, 2008) confirmed these results (Supplementary Dataset S2).

*IDA/IDL/PIP/PIPL* responses to hormone treatments were also studied using *in silico* data (Supplementary Fig. S4). Treatments with the ethylene precursor 1-aminocyclopropane-l-carboxylic acid (ACC), zeatin, abscisic acid (ABA), gibberellin A3 (GA-3) or brassinolide (BL) does not lead to any change in expression. *IDL7* is weakly induced by methyl jasmonate, whereas *PIPL3* and to a lesser extent *PIP2* are induced by indole-3-acetic acid (IAA).

**Fig. 8.**
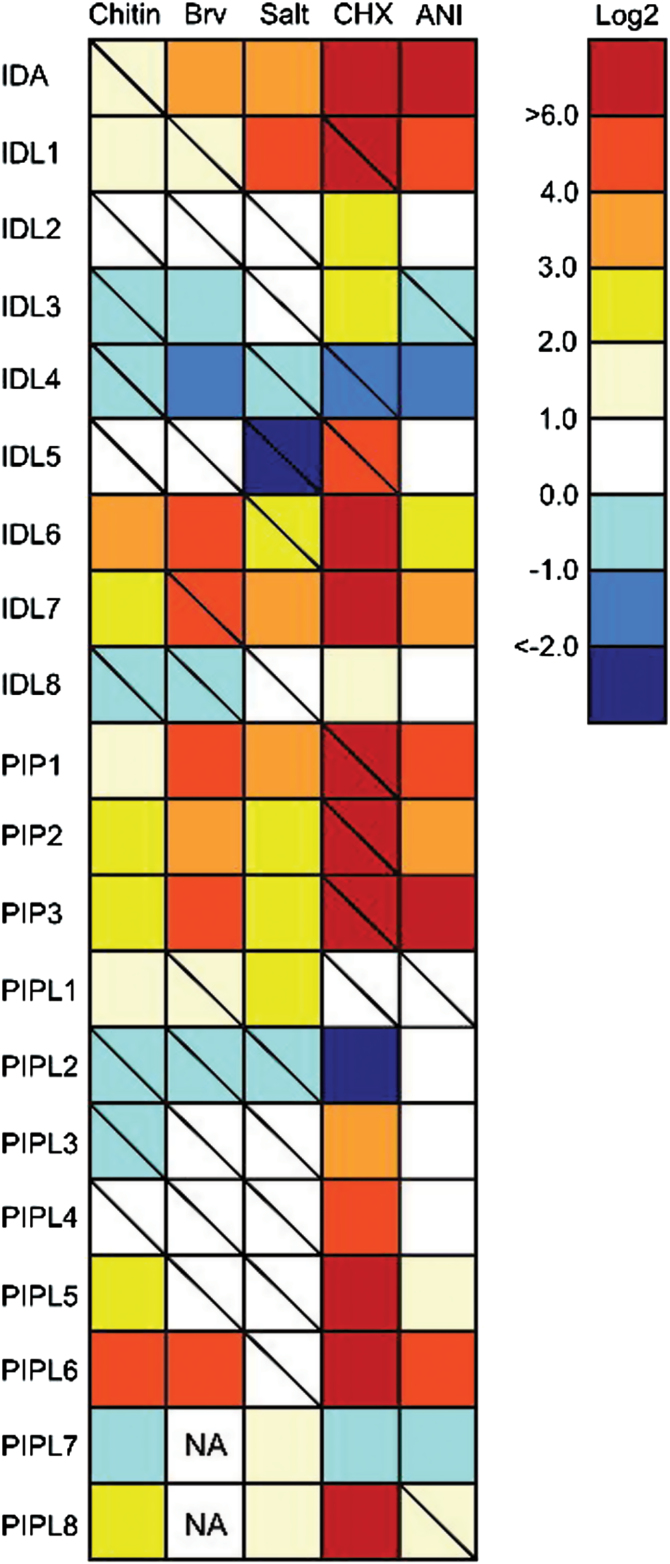
Stress-induced expression of *IDA/IDL* and *PIP/PIPL* genes based on qRT-PCR. Two-week-old plants were subjected to the following treatments for 6h unless otherwise specified: 10 μg/ml chitin (1h), 150mM NaCl, 10 μg/ml CHX and 15 μg/ml anisomycin. Infestation with *Brevicoryne brassicae* was done on 23-day-old plants, and rosette leaves were harvested after 72h (34). n=3. The relative expression (log2 ratios) values between treated and mock-treated samples are given next to the colour scale. Statistical differences between treated and mock-treated plants are indicated, where a diagonal line indicates no significance (REST analysis; P>0.05), and no diagonal line indicates significant difference (REST analysis; P<0.05). Brv, *Brevicoryne brassicae*; Salt, NaCl; CHX, cycloheximide; ANI, anisomycin.

In order to complement the *in silico* data, *Arabidopsis* Col-0 seedlings were subjected to various biotic and abiotic treatments and a qRT-PCR analysis was performed to obtain information on all *IDA/IDL* and *PIP/PIPL* members (Fig. 8). Treatments with the elicitor chitin induced several of the genes, with the highest log_2_ ratio observed for *IDL6* and *PIPL6*. The aphid *Brevicoryne brassicae*, which is a specialist on Brassicaceae species, was used as biotic treatment, and induced the expression of a large subset of the genes. Ten genes were up-regulated with log_2_>1; among these were *IDA, PIP2, PIP3* and *PIPL1*. Salt stress induced eight of the genes log_2_>1, confirming the *in silico* data.

CHX is a known inhibitor of protein synthesis (Schneider-Poetsch *et al.*, 2010). Interestingly, all the *IDL* genes present on the ATH1 array, as well as *PIP1*, *PIP2* and *PIP3*, are strongly induced upon CHX treatment (Fig. 7C). Treatment of seedlings with CHX led to an increase in the expression level of most of the *IDA/IDL/PIP/PIPL* genes 2-fold or more (Fig. 8). A subset of 12 genes showed an astounding response upon CHX treatment, with >1000-fold increase in expression levels. Generally, the genes found to be most inducible, either by abiotic or biotic stress, were strongly induced upon CHX treatment as well. These results were confirmed using another known protein synthesis inhibitor, anisomycin (Grollman, 1967). Similar to CHX, treatments with anisomycin highly induced the expression of ten of the *IDA/IDL/PIP/PIPL* genes (Fig. 8).

### Transcriptomic responses to PIPL3 peptide treatment

In order to investigate possible roles of IDA/IDL and PIP/PIPL peptides, the transcriptomic response of *Arabidopsis* seedlings to treatment with PIPL3 peptide was analysed. PIPL3 was chosen, as no functional data was available for this peptide; furthermore, *PIPL3* was expressed in leaf tissue during seedling stages.

**Fig. 9.**
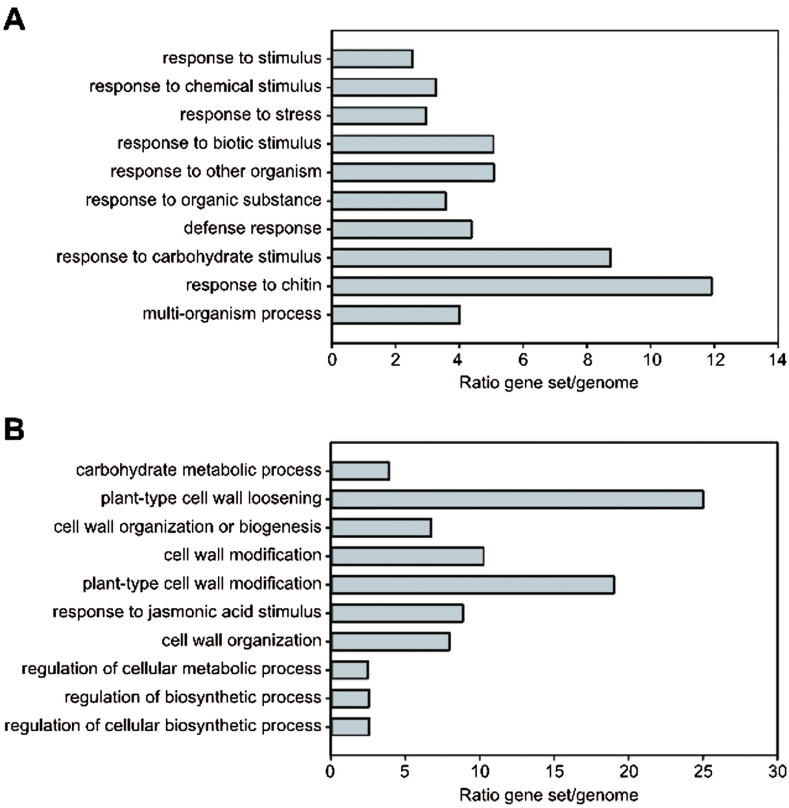
Transcriptional responses to PIPL3 peptide treatment. GO enrichment analysis of significantly regulated (*P*<0.05) genes in 2-week-old seedlings 3h after treatment with 100nM PIPL3 peptide. (A) Up-regulated genes; (B) Down-regulated genes. Control seedlings were treated with 100nM mock peptide. The dataset was filtered for expression ratio ratios (>log_2_ 1.0, <log_2_ −0.7). The 10 most significantly enriched terms are listed from top to bottom. The bars show the frequency ratios of each GO term in the PIPL3-responsive gene set versus the genome.

Treatment with 100nM PIPL3 peptide (containing a hydroxyproline in position 15) for 3h led to a widespread response compared with mock peptide-treated seedlings: 1599 genes were significantly (*P*<0.05) induced, whereas 1133 genes were significantly repressed. Genes showing low expression ratios (>log_2_ 1.0, <log_2_ −0.7) were removed, and the filtered dataset (291 induced and 129 repressed genes, respectively, Supplementary Dataset S3) was subjected to a Gene Ontology (GO) enrichment analysis (Ashburner *et al.*, 2000). The ten GO biological process categories most overrepresented in the up-regulated gene set indicated that the peptide treatment induced processes related to biotic stress and responses to chemical substances (possibly derived from other organisms) (Fig. 9A). The GO categories most enriched among the down-regulated genes were related to cell wall modification and loosening (Fig. 9B).

A more detailed analysis of the significantly regulated genes revealed that a majority of the genes encoding enzymes of the camalexin biosynthetic pathway were strongly induced by PIPL3 peptide treatment (Supplementary Fig. S5; Supplementary Dataset S3). Camalexin, an indole phytoalexin, is synthesized from tryptophan via indole-3-acetaldoxime (IAOx) (Glawischnig *et al.*, 2004; Ahuja *et al.*, 2012). IAOx is a branching point between the biosynthetic pathways of camalexin and two other groups of compounds: indole glucosinolates and IAA. Whereas the first biosynthetic components of the indole glucosinolate pathway were not transcriptionally responsive, the last steps, from indol-3-ylmethyl glucosinolate (I3M) to 4-methoxy-indol-3-ylmethyl glucosinolate (4MO-I3M), were induced (Supplementary Fig. S5). Indole glucosinolate and camalexin biosynthetic genes have been reported to be positively regulated by HIG1, WRKY33 and ANAC042, respectively (Gigolashvili *et al.*, 2007; Qiu *et al.*, 2008; Saga *et al.*, 2012). The expression of all three transcription factors was induced by PIPL3 treatment (Supplementary Dataset S3). In contrast, no genes encoding enzymes of the IAA biosynthetic pathway showed any response to PIPL3 treatment.

## Discussion

For the last decade, peptide ligands have been found to act as important regulatory factors in plants as well as in animal systems. A well-studied peptide is IDA, found to regulate floral organ abscission in *Arabidopsis* (Butenko *et al.*, 2003). In this study, we searched the *Arabidopsis* genome for genes encoding peptide ligands related to *IDA*, using both *in vivo* and *in silico* expression data to investigate these genes. In addition to three novel *IDL* genes, this search identified the recently described *PIP/PIPL* gene family. Of these genes, *IDL8* has not previously been annotated.

Our phylogenetic analyses indicate that the CEPs (Delay *et al.*, 2013; Roberts *et al.*, 2013) constitute a related peptide family, as they share the C-terminal GxGH motif with the PIP/PIPLs (Fig. 2A; Supplementary Fig. S1). CEP13, CEP14 and CEP15 might be considered to be a subclade of the CEP family, as suggested by Delay *et al.* (2013) and Roberts *et al.* (2013), or as a separate family. IDA/IDLs, PIP/PIPLs and CEPs all first appear in seed plants; the founder of these peptide families is therefore difficult to predict. A phylogenetic tree including IDA/IDL, PIP/PIPL and CEP members presented by Hou *et al.* (2014) differs topologically somewhat from ours (Supplementary Fig. S1). The tree produced by Hou *et al.* (2014) also includes members of the CLE and PEP families. Furthermore, it is based on an alignment of the C-terminal peptide motif, in contrast to the tree in Supplementary Fig. S1, which was generated from a full-length protein alignment. Thus, a direct comparison of these trees is difficult.

Families of post-translationally modified peptides are characterized by multiple paralogous genes encoding small, cysteine-poor peptides with high sequence diversity outside of the C-terminal domain, which contains the mature peptide (Matsubayashi, 2014). All *IDA/IDL* and *PIP/PIPL* family genes fulfil these criteria; they encode putative prepropeptides with an N-terminal SP followed by a variable sequence and a C-terminal, conserved motif related to the EPIP motif of IDA (Butenko *et al.*, 2003; Stenvik *et al.*, 2008). The highly conserved SGPS core motif contains a proline that is an attractive candidate for post-translational modification. Several characterized plant peptides, such as systemin (Pearce *et al.*, 1991), TRACHEARY ELEMENT DIFFERENTIATION INHIBITORY FACTOR (TDIF) (Ito *et al.*, 2006) and CEP1 (Ohyama *et al.*, 2008) have been shown to contain hydroxyproline. Furthermore, hydroxyprolines of PLANT PEPTIDE CONTAINING SULFATED TYROSINE 1 (PSY1) (Amano *et al.*, 2007), CLAVATA 3 (CLV3) and CLV3/EMBRYO SURROUNDING REGION-RELATED 2 (CLE2) (Ohyama *et al.*, 2009) are modified by the addition of an *O*-linked L-arabinose chain. These post-translational modifications increase the activity and/or specificity of the peptides (Shinohara and Matsubayashi, 2010, 2013).

We have previously noted that IDA/IDL peptides and several CLE peptides share a similar core with (hydroxy)prolines and small amino acids (Ala, Gly, Ser) (Stenvik *et al.*, 2008). These amino acids are also found in mature peptides of the PIP/PIPL and CEP families, and one may speculate that they are involved in receptor binding. Biochemical evidence has recently been provided for the binding of PIP1 to RLK7 (Hou *et al.*, 2014) and CEP peptides to CEPR1 and CEPR2 (Tabata *et al.*, 2014). Intriguingly, these three receptors are highly similar LRR-RLKs belonging to a subgroup of the LRR-RLK subclass XI that also includes HAE and HSL2 (Yamaguchi *et al.*, 2006; Butenko *et al.*, 2009). Sequence alignment (Supplementary Fig. S6) indicates that the LRR ectodomains of these three receptors are more similar to HAE and HSL2 than for instance to BAM1, which is documented to bind the CLE9 peptide (Shinohara *et al.*, 2012). Other members of the HAE/HSL2 branch of LRR-RLKs should be promising candidate receptors for peptides belonging to the IDA/IDL, PIP/PIPL and CEP families.

Interestingly, PIP2, PIP3 and PIPL1 apparently possess two SGPS motifs (Fig. 1; Hou *et al.*, 2014). The double peptide motif might be processed into two independent peptides that may bind different receptor complexes, thus activating different pathways. As a result, the response induced by the peptides may be wider and/or stronger. Alternatively, the double peptide acts as one functional unit that may interact with two different binding sites, or even to different partners in a receptor dimer. Multiple peptide motifs have also been identified in members of the CEP family (Delay *et al.*, 2013; Roberts *et al.*, 2013). A member of the CLE family, *CLE18,* encodes a precursor protein that contains two functional peptide motifs. The 13 aa CLE18 peptide located in the CLE18 variable region inhibits tracheary element differentiation and suppresses root growth (Ito *et al.*, 2006), whereas the newly discovered C-terminal 12 aa CLE-LIKE peptide motif, promotes root growth (Meng *et al.*, 2012), suggesting that one gene can encode two peptides with different roles. Crosstalk between different pathways is an important way to fine-tune the response to a given signal, and is normally mediated by common components in signalling pathways (Knight and Knight, 2001; Fujita *et al.*, 2006). The double peptides motifs of PIP2, PIP3 and PIPL1 might provide plants with an extra dimension in crosstalk; one gene encodes two possible peptides that may modulate two different pathways.

Many of the *IDA/IDL/PIP/PIPL* genes have tissue-specific expression (Figs 4–6), suggesting that these genes may play roles during plant growth and development (Stenvik *et al.*, 2008). We were hardly able to detect expression of *IDA*, *IDL1*, *IDL2* and *IDL5* in different organs during development (Fig. 5), but as shown in Fig. 6, expression of *IDA* and *IDL1– IDL5* is restricted to very specific tissues or cell types, indicating a strict developmental regulation of transcription. This is further confirmed by the abiotic and biotic stress assays (Fig. 8), where *IDL2– IDL5* appear to be non-responsive. IDA is strongly regulated by IAA in roots (Kumpf *et al.*, 2013), but our analysis of *in silico* data suggests that this is not the case for green tissue (Supplementary Fig. S5; Supplementary Dataset S2). *PIPL1* shows the highest expression levels of the 20 genes in our *in silico* analysis (Fig. 4), but as for the *IDL* genes, the expression is too specific to be detected in our qRT-PCR analysis (Fig. 5). Transcriptome studies of *Arabidopsis* seed development indicate that *PIPL1* expression is restricted to seed coat tissue during early stages of seed development (Le *et al.*, 2010; Belmonte *et al.*, 2013).

Stress-induced genes (Figs 7, 8) include *PIP1, PIP2, PIP3, PIPL5* and *PIPL6*, while *IDA, IDL1, IDL6* and *IDL7* are up-regulated both during development and stress. The stress-induced genes can be separated into two categories: those induced by abiotic stress (like *IDA, IDL1, PIP1*) and those induced by biotic stress (like *IDL6*). *IDL7, PIP2* and *PIP3* are induced by both abiotic and biotic stress. PIP1 and PIP2 have been implicated in immune responses and pathogen resistance (Hou *et al.*, 2014). Our results suggest that IDA/IDL and PIP/PIPL peptides also may be involved in regulation of responses to abiotic stresses such as salt stress. The peptides could act in positive or negative feedback loops for temporal and/or spatial fine-tuning of stress signalling pathways.

A subset of the *IDA/IDL* and *PIP/PIPL* genes were strongly induced by treatment with the translational inhibitors CHX and anisomycin (Figs 7, 8). Such superinduction has previously been reported in plants, for cold-induced genes (Berberich and Kusano, 1997; Zarka *et al.*, 2003) and immediate-early response genes (Horvath *et al.*, 1998; Uquillas *et al.*, 2004), amongst others. The mechanism behind CHX superinduction could be related to the presence of a labile transcriptional repressor or increased mRNA stability.

Treating seedlings with PIPL3 peptide led to the induction of genes involved in defence responses, including the camalexin and indole glucosinolate biosynthesis pathways (Supplementary Fig. S5). Neither *in silico* data (Fig. 7; Supplementary Fig. S3) nor our own experiments (Fig. 8) indicate that *PIPL3* expression is activated by biotic or abiotic treatments; instead, expression appears to be auxin-induced (Supplementary Fig. S4; Supplementary Dataset S2). However, PIP1 and PIP2, the other family members for which functional data exist, have been strongly implicated as positive regulators of defence responses (Hou *et al.*, 2014). Both camalexin and indole glucosinolates are important parts of the chemical defence system of crucifers (Sønderby *et al.*, 2010; Ahuja *et al.*, 2012). Whereas glucosinolates are stored after synthesis and are detected in most tissues (Brown *et al.*, 2003), camalexin is produced upon biotic or abiotic stress (Glawischnig, 2007). Why are these pathways induced by a peptide that shows developmentally regulated expression? It is tempting to speculate that PIPL3 is involved in regulation of the trade-off between growth and defence during development. PIPL3 may activate a limited set of defence-related pathways to a basal expression level in tissue surrounding meristematic regions. The down-regulation of cell wall loosening genes by PIPL3 indicate that the target cells could be differentiated cells that have reached their final size and shape.

In this study, we have characterized the IDA/IDL and PIP/PIPL families of peptide ligands in *Arabidopsis*. The family is characterized by one or both of two C-terminal conserved peptide motifs, SGPS and GxGH. Three PIP/PIPL members contain two tandem peptide motifs. Members of the *IDA/IDL* and *PIP/PIPL* gene families are expressed during development or induced by stress, suggesting distinct biological roles. Transcriptome analysis of PIPL3 peptide-treated seedlings indicates that although *PIPL3* expression appears to be developmentally regulated, it activates defence-related processes.

## Supplementary data

Supplementary data are available at *JXB* online.

Supplementary Figure S1. Phylogenetic relationship between IDA/IDL, PIP/PIPL and CEP peptides in *Arabidopsis*.

Supplementary Figure S2. Chromosomal localization of *Arabidopsis IDA/IDL* and *PIP*/*PIPL* genes.

Supplementary Figure S3. Elicitor-induced expression of *IDA/IDL* and *PIP*/*PIPL* genes based on *in silico* data.

Supplementary Figure S4. Transcriptional responses of *IDA/IDL* and *PIP/PIPL* genes to hormone treatments.

Supplementary Figure S5. Transcriptional activation of the camalexin and indole glucosinolate biosynthetic pathways by PIPL3 peptide treatment.

Supplementary Figure S6. IDA, PIP1 and CEP1 bind related proteins.

Supplementary Table S1. List of PCR primers used in this study.

Supplementary Table S2. Gene duplication events of the *IDA/IDL* and *PIP/PIPL* gene families.

Supplementary Table S3. Expression of *IDA* and the *IDL1* to *IDL5* genes fourteen days after germination.

Supplementary Dataset S1. Protein sequences of IDA/IDL and PIP/PIPL in *Arabidopsis*.

Supplementary Dataset S2. Transcriptional responses of *IDA/IDL/PIP/PIPL* genes to perturbations, based on *in silico* data from Genevestigator.

Supplementary Dataset S3. Transcriptome responses to PIPL3 peptide treatment.

Supplementary Data
