# Supplementary material for: The IDA/IDA-LIKE and PIP/PIP-LIKE gene families in Arabidopsis: phylogenetic relationship, expression patterns, and transcriptional effect of the PIPL3 peptide
Source: J Exp Bot. 2015 Jun 10;66(17):5351–65. doi: 10.1093/jxb/erv285 (PMC4526919; doi:10.1093/jxb/erv285)
Supplement: Supplementary Data [file supp_66_17_5351__index.html]

The IDA/IDA-LIKE and PIP/PIP-LIKE gene families in Arabidopsis: phylogenetic relationship, expression patterns and transcriptional effect of the PIPL3 peptide — The IDA/IDA-LIKE and PIP/PIP-LIKE gene families in Arabidopsis: phylogenetic relationship, expression patterns, and transcriptional effect of the PIPL3 peptide — Supplementary Data 

# The *IDA/IDA-LIKE* and *PIP/PIP-LIKE* gene families in *Arabidopsis*: phylogenetic relationship, expression patterns, and transcriptional effect of the PIPL3 peptide

## Supplementary Data

Data files

- Supplementary Data - Supplementary Data
- Supplementary Data - Supplementary Data
- Supplementary Data - Supplementary Data
