# Supplementary material for: The IDA/IDA-LIKE and PIP/PIP-LIKE gene families in Arabidopsis: phylogenetic relationship, expression patterns, and transcriptional effect of the PIPL3 peptide
Source: J Exp Bot. 2015 Jun 10;66(17):5351–65. doi: 10.1093/jxb/erv285 (PMC4526919; doi:10.1093/jxb/erv285)
Supplement: Supplementary Data [file supp_erv285_jexbot148056_file003.pdf]

The IDA/IDA-LIKE and PIP/PIP-LIKE gene families in Arabidopsis: phylogenetic relationship, expression patterns and transcriptional effect of the PIPL3 peptide

Ane Kjersti Vie, Javad Najafi, Bin Liu, Per Winge, Melinka A. Butenko, Karina S. Hornslien, Robert Kumpf, Reidunn B. Aalen, Atle M. Bones and Tore Brembu

Supplementary Data

Figs S1-S6
Table S1-S3
Dataset S1

Supplementary Figure S1

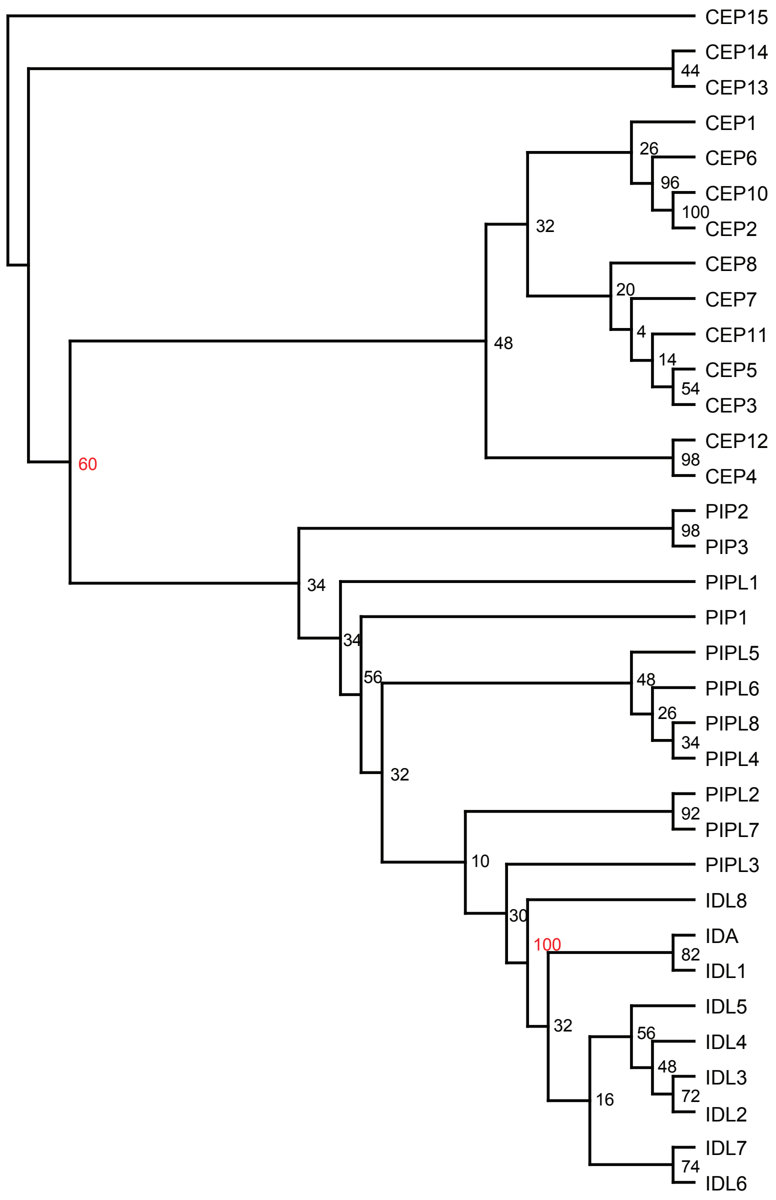

Supplementary Figure S1. Maximum Likelihood (ML) tree constructed based on a full-length protein alignment of the Arabidopsis IDA/IDL, PIP/PIPL and CEP families. CEP15 was used as an outgroup. CEP9 was not included, due to its aberrant length (229 AAs) and number of peptide motifs (five). Bootstrap confidence values are shown in percentage. Nodes discussed in the text are shown in red.

Supplementary Figure S2

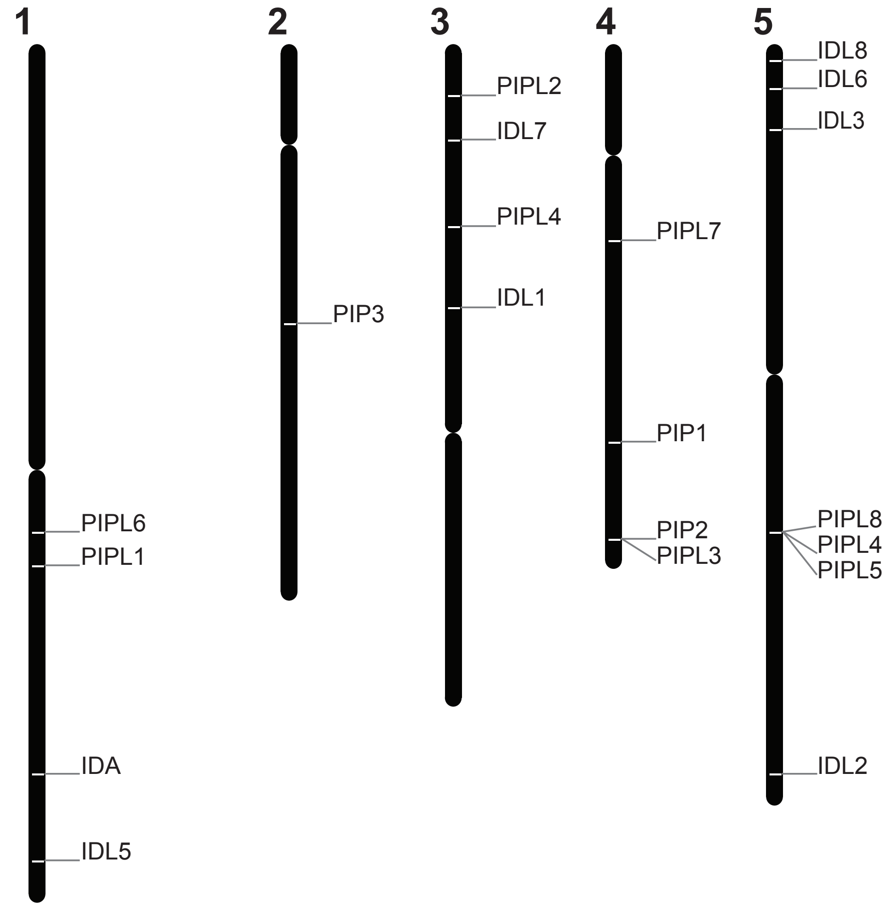

Supplementary Figure S2. Chromosomal localisation of *IDA*/*IDL* and *PIP/PIPL* genes in Arabidopsis. The chromosome number is indicated above each chromosome.

Supplementary Figure S3

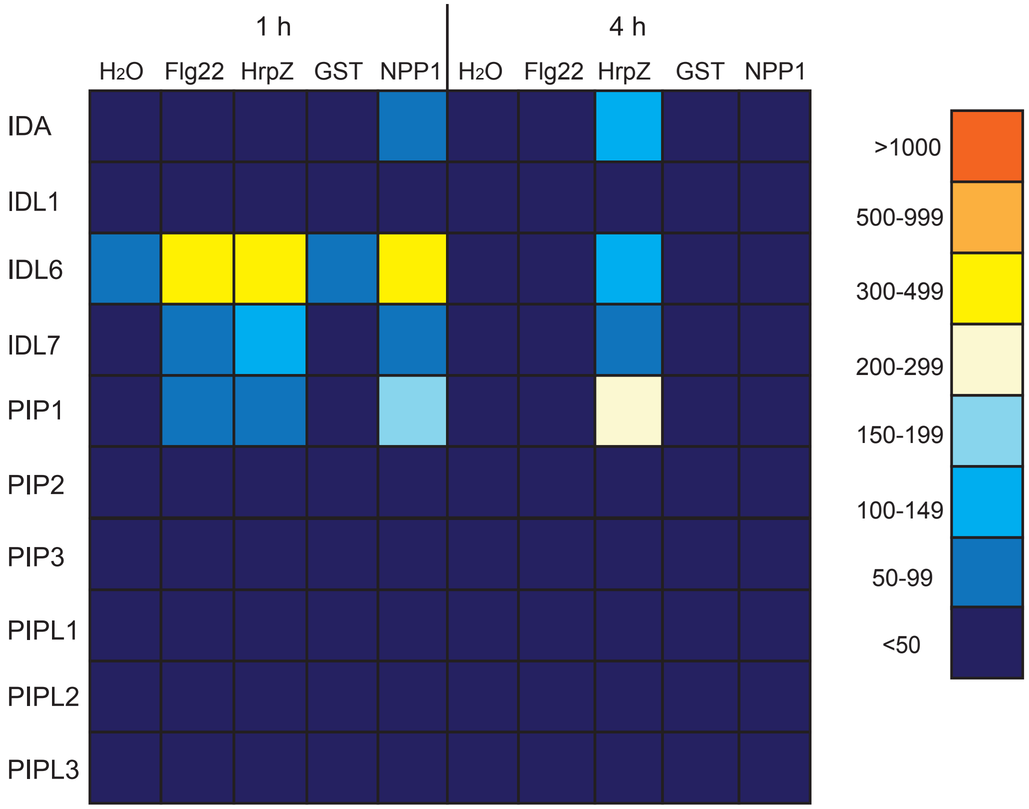

Supplementary Figure S3. Elicitor-induced expression of *IDA/IDL* and *PIP/PIPL* genes based on *in silico* data.

H2O, Control plants treated with water; Flg22; flagellin22 (*Pseudomonas syringae* derived elicitor); HrpZ, HrpZ hairpin (*Pseudomonas syringae* derived elicitor); GST, Control plants treated with Gluthatione S-transferase (GST) tag; NPP1, Necrotic-inducing Phytophthora protein 1, GST-tagged (*Phytophthora infestans* derived elicitor). All data was obtained from the Arabidopsis eFP browser at the Bio-Array Resource database (Winter et al., 2007). The arithmetic expression values are given next to the colour scale.

Supplementary Figure S4

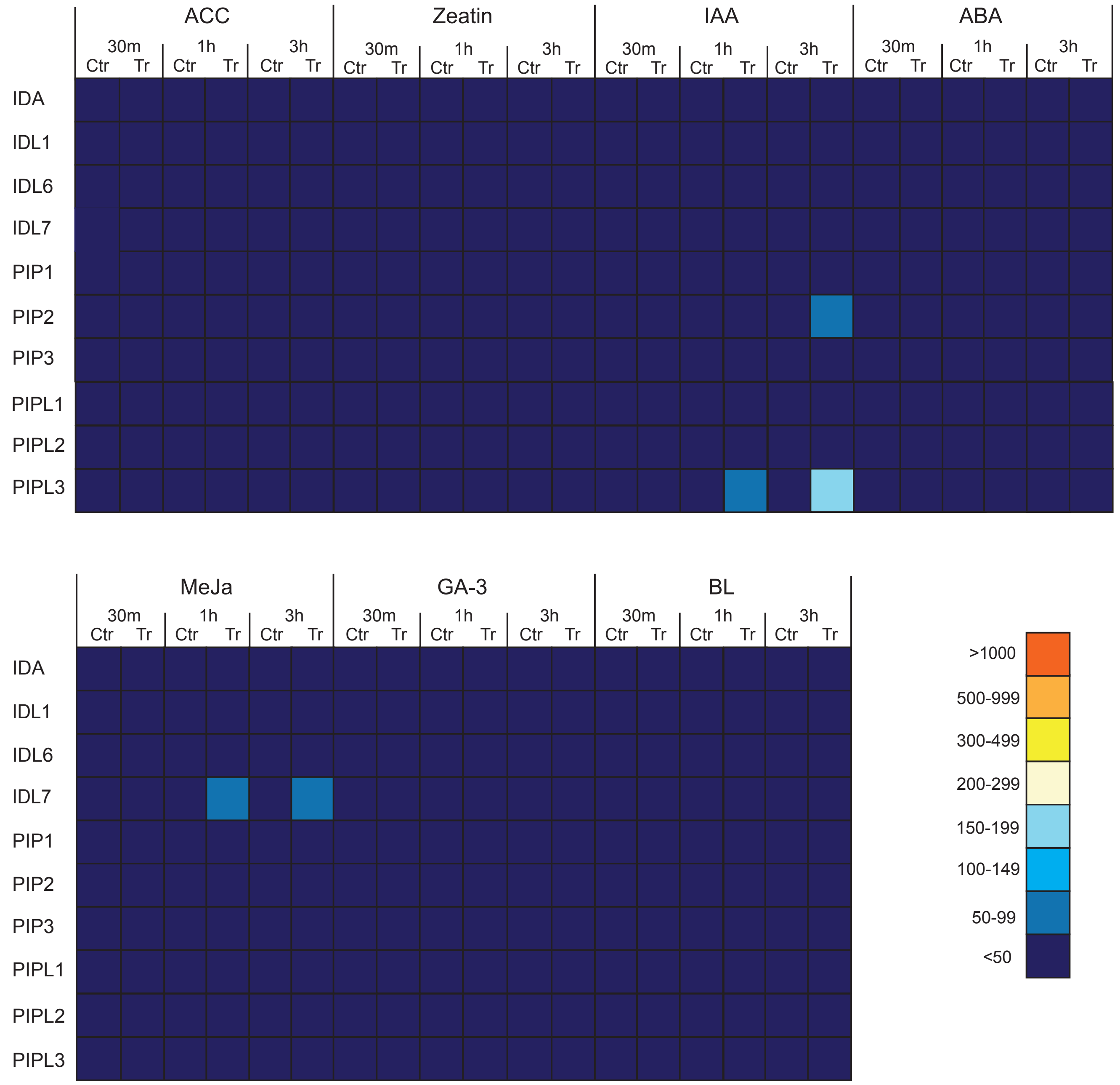

Supplementary Figure S4. Hormone-induced expression of *IDA/IDL* and *PIP/PIPL* genes based on *in silico* data. ACC, 1-aminocyclopropane-l-carboxylic acid; ABA, abscisic acid; BL, brassinolide; GA-3, gibberellin A3; IAA, indole-3-acetic acid; MeJa, methyl jasmonate. All data was obtained from the Arabidopsis eFP browser at the Bio-Array Resource database (Winter et al., 2007). The arithmetic expression values are given next to the colour scale.

Supplementary Figure S5

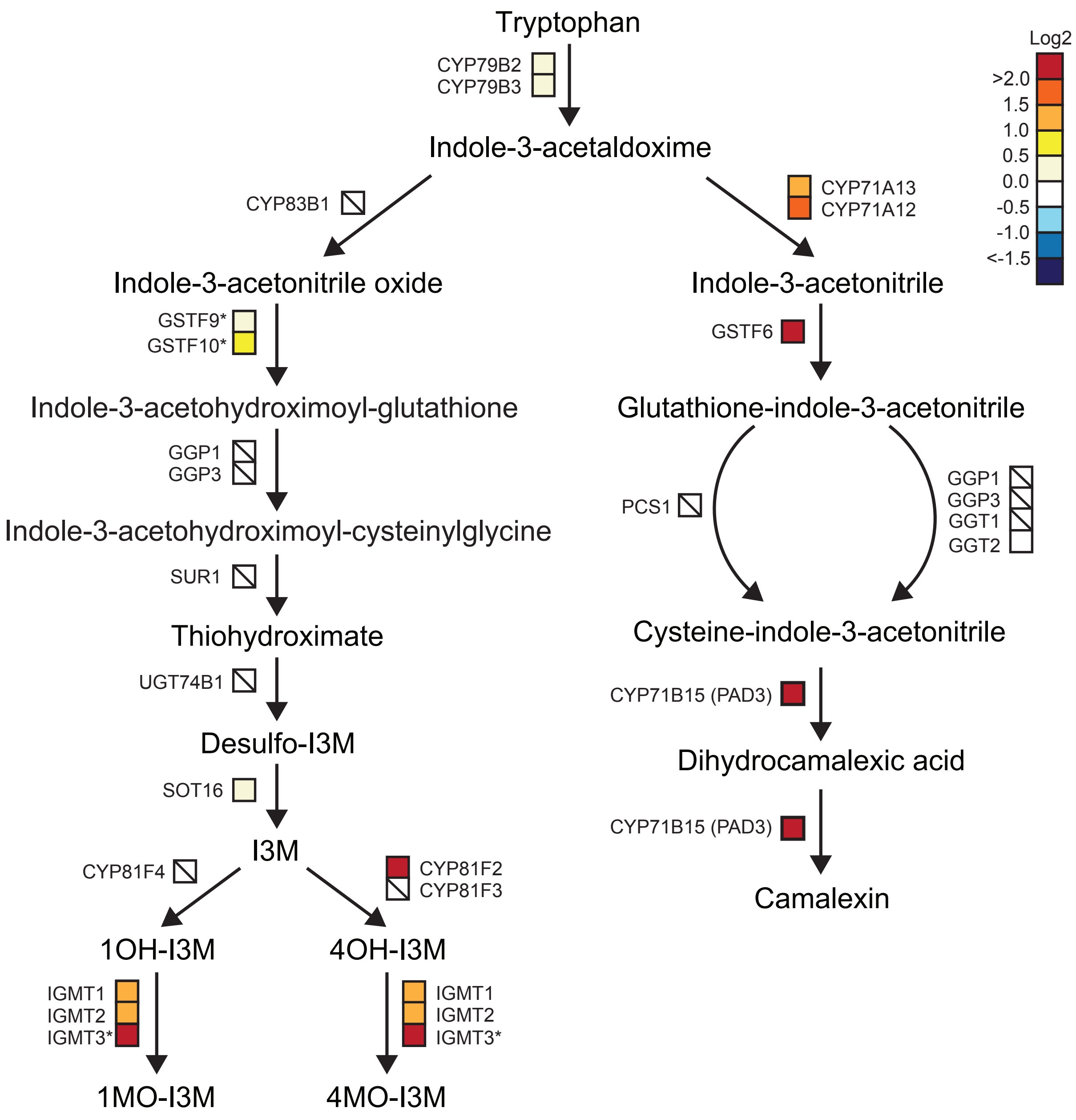

Supplementary Figure S5. Transcriptional activation of the camalexin and indole glucosinolate biosynthetic pathways by PIPL3 peptide treatment. Squares with a diagonal line inside indicate genes that are not significantly regulated. The scale on the right represents gene expression ratio values, log2 transformed. 1MO-I3M, 1-methoxy-indole-3-ylmethyl glucosinolate; 1OH-I3M, 1-hydroxy-indol-3-yl-methyl-glucosinolate; 4MO-I3M, 4-methoxy-indol-3-ylmethyl glucosinolate; 4OH-I3M, 4-hydroxy-indole-3-ylmethyl-glucosinolate; I3M, indol-3-ylmethyl glucosinolate. Asterisks indicate predicted enzymes. The figure is adapted from Sønderby et al., 2010 and Ahuja et al., 2012.

Supplementary Figure S6

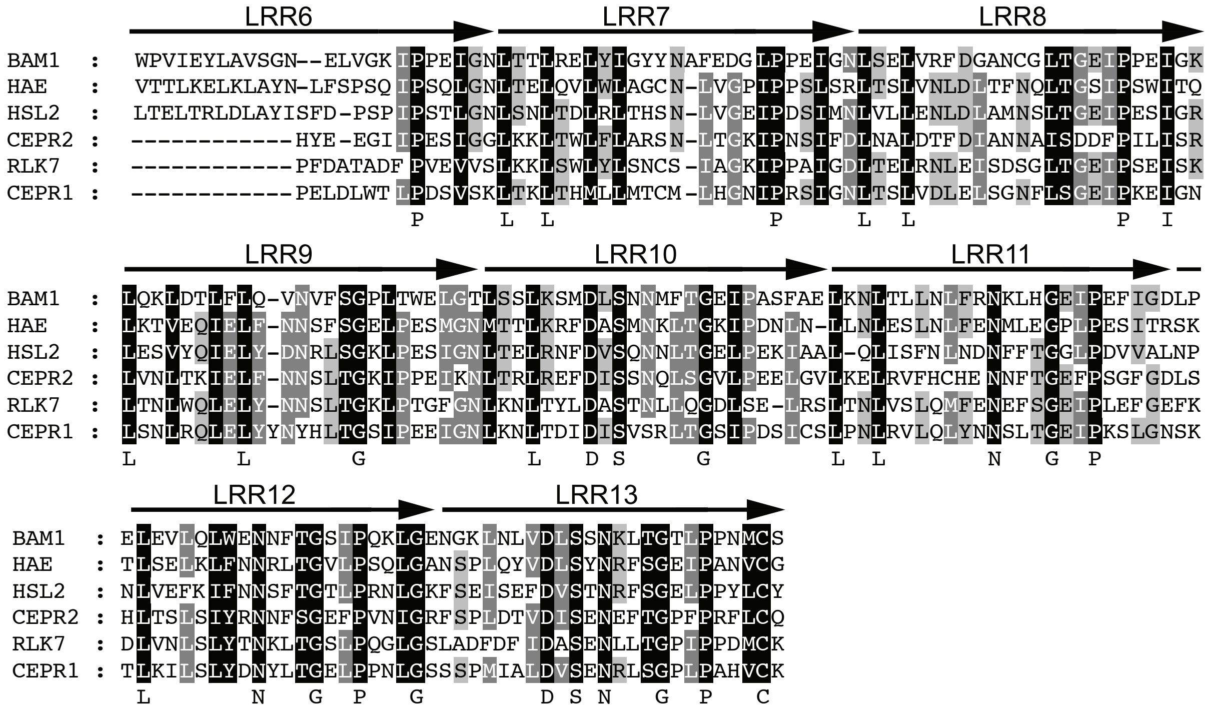

Supplementary Figure S6. IDA, PIP1 and CEP1 bind related receptors. Protein alignment of a central part of the ectodomains of the receptors showing the LRRs 6-13. Repeats 6-8 of BAM1 has been shown to be involved in the binding of CLE9 (Shinohara *et al.*, 2012). Amino acids in black are conserved in all sequences, consensus amino acids are shown below the alignment.

Supplementary Table S1. List of primers used in this study.

Gene	AtID	Primer	Sequence
IDA	At1g68765	qIDAF	CAATGGCTCCGTGTCGT
		qIDAR	TCAATGAGGAAGAGAGTTAACAAAAGAG
IDL1	At3g25655	qIDL1F	AAAATCCAAAATTTATAAAACCCTAGC
		qIDL1R	AAACATGGTTTTATGAGAAAGATTCA
IDL2	At5g64667	qIDL2F	ATTCAGCAATCTCCTTTTTCCC
		qIDL2R	TTTGTGGAAGGGTTTTTGGTGA
IDL3	At5g09805	qIDL3F	CCTGCCACGTCAGTTTCC
		qIDL3R	CCGATTTCGTTATGTTTTCTGG
IDL4	At3g18715	qIDL4F	GCGGAGAGGCCATTTCTTA
		qIDL4R	AGGAGCAGAAGCTGGAACAG
IDL5	At1g76952	qIDL5F	TCAGGATTTTTGCCCAAGAC
		qIDL5R	GCTCGACCTTGAGTTGGTTCT
IDL6	At5g05300	qIDL6F	TGCTTCGTTCTCAACAGCTAGG
		qIDL6R	GAATATCCAGCCGTCAAGTGAT
IDL7	At3g10930	qIDL7F	CCGGAGAGTTTGTTCCAGTCAT
		qIDL7R	CGTTAGTTTTCTTGCTGGGTCC
IDL8	At5g02591[a]	qIDL8F	ATAAACCAACCATCTTCGGCTT
		qIDL8R	ACGAGTATCATACGGATCTAGG
PIP1	At4g28460	qPIP1F	CGATGAGAAGAGTTAGTTGGTC
		qPIP1R	GGAACCACCACGTGTTCTACGA
PIP2	At4g37290	qPIP2F	TTAAAGTGCCCATACCCATCTA
		qPIP2R	TCTTCCTATGTATCGATCAGAA
PIP3	At2g23270	qPIP3F	GACTGAGACGCTTGAATATGGT
		qPIP3R	AAGCAGCCACATTAACTGGTTC
PIPL1	At1g49800	qPIPL1F	GTCAAGGATAGTTCGAGATCGT
		qPIPL1R	GACTGTTTCACTGTGGCTAACA
PIPL2	At3g06090	qPIPL2F	CATGGTTGTAACAGGGTTGCAG
		qPIPL2R	TTCGACCTGACCACAAATTCGC
PIPL3	At4g37295	qPIPL3F	ATGCATACAATGGCTTCAGGAC
		qPIPL3R	GATTCTATGACCTGAGCTAAAC
PIPL4	At5g43066	qPIPL4F	GGAGCCTTTCAGAGATATCAAC
		qPIPL4R	CAGACGCTAGAATCCTCATCAA
PIPL5	At5g43068[a]	qPIPL5F	CTCTACGATCAAAGGCGTTGAG
		qPIPL5R	TCTTCCCCAAAGGACGTTGACT
PIPL6	At1g47178[a]	qPIPL6F	CTAATCAGAGGCTTAGTTACTG
		qPIPL6R	ATGTATAACGACTTGTGCAAAC
PIPL7	At5g43066[a]	qPIPL7F	GTACCATATCGTGGTTGTGACG
		qPIPL7R	CGACTCGGACCAGAAACTAACT
PIPL8	At4g11403[a]	qPIPL8F	TTGAGGGTGGTCATTGCAGAAC
		qPIPL8R	GTTGGTATCTCTGAACGGCTTC
Cyp71A13	At2g30770	qCYP71A13F	TAAAGAGGTGCTTCGGTTGC
		qCYP71A13R	TATCGCAGTGTCTCGTTGGA
TIP41-LIKE	At4g34270	qTIP41-likeF	GTGAAAACTGTTGGAGAGAAGCAA
		qTIP41-likeR	TCAACTGGATACCCTTTCGCA

[a]Preliminary AtID from Tair.

Supplementary Table S2. Gene duplication events of the *IDA/IDL and PIP/PIPL* gene families. The 40 nearest protein coding genes flanking each of the *IDA/IDL/PIP/PIPL* gene loci were analysed for other closely related genes located next to the *IDA* and *PIP/PIPL* genes by Blast searches. Each number represents common genes flanking the *IDA/IDL/PIP/PIPL* loci.

	IDA	*IDL1*	*IDL2*	*IDL3*	*IDL4*	*IDL5*	*IDL6*	*IDL7*	*IDL8*	*PIP1*	*PIPL6*	*PIPL5-PIPL4-PIPL8*[a]	*PIPL2*	*PIPL7*	*PIPL3-PIP2*[a]	*PIP2*	*PIPL1*
IDA	0	7	0	0	0	0	0	0	0	1	0	0	1	0	0	0	0
IDL1	7	0	0	0	0	0	0	0	0	1	0	0	0	0	0	0	0
IDL2	0	0	0	14	1	0	1	0	0	0	1	0	0	0	1	0	0
IDL3	0	0	15	0	1	0	1	1	0	0	0	0	0	0	1	0	0
IDL4	2	2	1	3	0	0	1	0	0	0	0	0	0	1	0	0	0
IDL5	0	0	0	0	0	0	0	0	0	0	0	0	0	0	0	0	0
IDL6	0	0	0	0	2	0	0	6	0	0	0	0	1	0	0	0	0
IDL7	0	0	0	1	0	0	6	0	0	0	0	0	0	1	0	0	0
IDL8	0	0	0	0	0	0	0	0	0	0	0	0	0	1	0	0	0
PIP1	0	1	2	1	0	0	0	2	0	0	0	0	0	0	0	0	0
PIPL6	0	0	1	0	0	0	0	0	0	0	0	5	0	0	0	2	0
PIPL5-PIPL4-PIPL8[a]	0	0	1	1	0	0	0	0	0	0	5	0	0	0	0	0	1
PIPL2	0	0	0	0	0	0	0	0	0	0	0	0	0	0	0	0	0
PIPL7	0	0	0	0	0	0	0	1	0	0	2	2	0	0	0	0	0
PIPL3-PIP2[a]	1	0	1	1	0	0	0	0	0	0	0	0	0	0	0	4	0
PIP2	0	0	0	0	0	0	0	0	0	0	2	0	0	0	8	0	1
PIPL1	0	0	0	0	0	0	0	0	1	0	0	0	0	0	0	1	0

[a] *PIPL4*, *PIPL5* and *PIPL8* are localised next to each other on chromosome 5 and are therefore treated as one locus. The same is the situation for *PIPL3* and *PIP2* on chromosome 4

Supplementary Table S3. Expression of *IDA* and the *IDL1 to IDL5* genes fourteen days after germination. Presence of a GUS signal in different tissues of the developing seedling. (-) indicates no *GUS* expression, the location of the GUS signal is indicated by tissue.

	**Cotyledons**	**Roots**	**Hypocotyl**	**Stomata**	**Shoot apical meristem**
IDA	-	cortex/epidermis	-	-	-
IDL1	vascular	columella	-	-	+
IDL2	vascular	vascular	-	+	+
IDL3	vascular	vascular	vascular	+	+
IDL4	vascular	vascular	vascular	-	+
IDL5	-	-	-	-	-

Supplementary Dataset 1. Protein sequences of IDA family members in Arabidopsis.
[a]Preliminary AtID from TAIR

>IDA_ At1g68765
MAPCRTMMVLLCFVLFLAASSSCVAAARIGATMEMKKNIKRLTFKNSHIFGY
LPKGVPIPPSAPSKRHNSFVNSLPH

>IDL1_ At3g25655
MNLSHKTMFMTLYIVFLLIFGSYNATARIGPIKLSETEIVQTRSRQEIIGGFTFK
GRVFHSFSKRVLVPPSGPSMRHNSVVNNLKH

>IDL2_ At5g64667
MSSRNQRSRITSSFFVSFFTRTILLLLILLLGFCNGARTNTNVFNSKPHKKHND
AVSSSTKQFLGFLPRHFPVPASGPSRKHNDIGLLSWHRSSP

>IDL3_ At5g09805
MSSRSHRSRKYQLTRTIPILVLLLVLLSCCNGARTTNVFNTSSPPKQKDVVSPP
HDHVHHQVQDHKSVQFLGSLPRQFPVPTSGPSRKHNEIGLSSTKT

>IDL4_ At3g18715
MYPTRPHYWRRRLSINRPQAFLLLILCLFFIHHCDASRFSSSSVFYRNPNYDHS
NNTVRRGHFLGFLPRHLPVPASAPSRKHNDIGIQALLSP

>IDL5_ At1g76952
MGNKRIKAMMILVVMIMMVFSWRICEADSLRRYSSSSRPQRFFKVRRPNPRN
HHHQNQGFNGDDYPPESFSGFLPKTLPIPHSAPSRKHNVYGLQSTNSHRCP

>IDL6_ At5g05300
MARIGALILVLFISISQLASFSTARKFPVGIPSVIDGVIFSGEISAVSKKVTVVGC
EGEDDHLTAGYSSYITGKFGSLVLNALPKGSVPASGPSKRINDVKT

>IDL7_ At3g10930
MAINRSLLLLILLFISVSLSTARILPGEFVPVIFSGEIPPVSKSAVVGCGGEQETKT
EYSSFVPEVVAGKFGSLVLNALPKGSRPGSGPSKKTNDVKT

>IDL8_ At5g02591[a]

MAKSTYVLVVISFGLLFACVIGTTQDETSRLLWSRPWARGLADSPPQDPHKPT
IFGLKPWSPSQRLIFRMLPKNVPIPPSGPSRKETPPSPPRSV

>PIP1_ At4g28460

MRRVSWSTVLIVVVMVSLFFVEHVVVPAAAGRVLTEKSGDGSATMTVEKM
KSTVDSWFQRLASGPSPRGRGH

>PIP2_ At4g37290

MMMNKNVLSSILFFMLIGSVLVESRPLGLTKTEEKFVASLFDGLSLGSIKDSGP
SPGEGHKVVDRKDTFRFVKHSGPSPSGPGH

>PIP3_ At2g23270

MMMNKVVLGSFLFFMLVSSNFVVEARPLGLTKAEENLVAKFFDGLSLGAIKE
SGPSSGGEGHRFVDRTETLEYGKHSGPSTSGPGH

>PIPL1_ At1g49800

MVMAKNLTKFYVVFLVVLMMVVSLLLAIEGRPVKDSSRSLTQMRDSSMFNG
SVIMSSFKPVESSVKDLSWLATVKQSGPSPGVGHHRAKGYKMFGRANDSGPS
PGVGH

>PIPL2_ At3g06090

MKMKKLLKVVFLLVAYLTCSIAMASYHGCNRVAEKATRMNVVGEDSRNEF
GNYEEKKKKANLWSGRKLASGPSRRGCGH

>PIPL3_ At4g37295

MRPVGLIFTVMFLVSAFSESRTADCRVLLGGSTEEIDQSKIHGVDLRSEDLLG
VVIHGYKKLRWLSSAGERMHTMASGPSRRGAGH

>PIPL4_ At5g43066

MEKKNMFVLCMILLLVGSSLMFERVDCRVVRSEPFRDINGHDQSTATKVKRS
SCSRRPLMRILASGPNKRGRGH

>PIPL5_ At5g43068[a]

MEKKNVFLLFVIFLLVGSSLMFERVDCRALRSKALRDIKGHDQSSVIMKVKN
NSTSQRPLGKSFAYRLASGPSRRGRGH

>PIPL6_ At1g47178[a]

MEKKLMILLCIIFLVEVFSILGGVDCRALRTQTTGCDQTTGCDQTTATVESGG
FGVFSGAANGSRDHLLMRSLAFRLASGPSRKGRGH

>PIPL7_ At4g11402[a]

MEMKTLLKVMFFLFSYLSTFSLAMVPYRGCDVIGVDLGDGSGNYGKGVSKG
FLSGRKLVSGPSRSSCGH

>PIPL8_ At5g43064[a]

MENKNVFVLCVILFLVSSSLMFEGGHCRTLRSKPFRDTNGHDQSSATMKVKH
SSESFAFRLASGSSRRGRGH
